# Introducing SpectraFit: An Open-Source Tool for Interactive Spectral Analysis

**DOI:** 10.1021/acsomega.3c09262

**Published:** 2024-05-20

**Authors:** Anselm W. Hahn, Joseph Zsombor-Pindera, Pierre Kennepohl, Serena DeBeer

**Affiliations:** † Max Planck Institute for Chemical Energy Conversion, Stiftstraße 34-36, Mülheim an der Ruhr 45470, Germany; ‡ Department of Chemistry, University of Calgary, Calgary, AB T2N 1N4, Canada; § Department of Chemistry, The University of British Columbia, Vancouver, BC V6T 1Z1, Canada

## Abstract

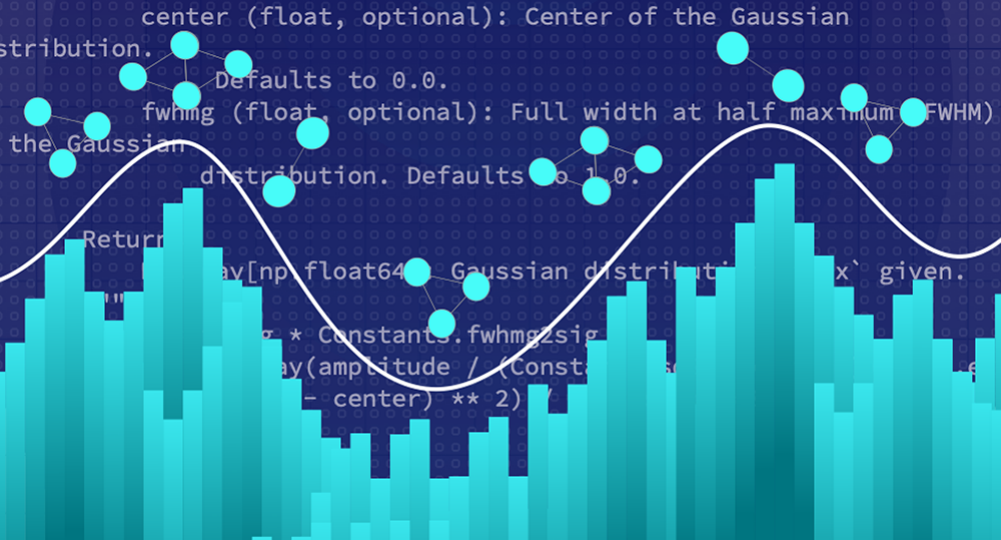

In chemistry, analyzing spectra through peak fitting is a crucial task that helps scientists extract useful quantitative information about a sample’s chemical composition or electronic structure. To make this process more efficient, we have developed a new open-source software tool called SpectraFit. This tool allows users to perform quick data fitting using expressions of distribution and linear functions through the command line interface (CLI) or Jupyter Notebook, which can run on Linux, Windows, and MacOS, as well as in a Docker container. As part of our commitment to good scientific practice, we have introduced an output file-locking system to ensure the accuracy and consistency of information. This system collects input data, results data, and the initial fitting model in a single file, promoting transparency, reproducibility, collaboration, and innovation. To demonstrate SpectraFit’s user-friendly interface and the advantages of its output file-locking system, we are focusing on a series of previously published iron–sulfur dimers and their XAS spectra. We will show how to analyze the XAS spectra via CLI and in a Jupyter Notebook by simultaneously fitting multiple data sets using SpectraFit. Additionally, we will demonstrate how SpectraFit can be used as a black box and white box solution, allowing users to apply their own algorithms to engineer the data further. This publication, along with its Supporting Information and the Jupyter Notebook, serves as a tutorial to guide users through each step of the process. SpectraFit will streamline the peak fitting process and provide a convenient, standardized platform for users to share fitting models, which we hope will improve transparency and reproducibility in the field of spectroscopy.

## Introduction

In the field of chemistry, fitting data is a standard part of the workflow.^1−7^ The data can come from either experiments^1,4,5,7^ or theoretical models,^6,8^ but being able to process and quantitatively analyze the data is crucial in order to reliably draw conclusions. This is especially true in spectroscopy, where fitting is necessary to match experimental peaks to a quantum chemical or semiempirical model.^1,4,9^ However, the process of fitting can be complicated and time-consuming, and finding ways to streamline these processes could significantly enhance workflow efficiency.^2,10^

Here, we see two opposite approaches to fitting spectra. On the one hand, there are the so-called “black-box”^11^ fitting approaches, which allow users to fit spectra with a few clicks and are often implemented in commercial software like Origin or IgorPro. On the other hand, there are the “white-box”^11,12^ fitting approaches, where users usually have to program their own individual fitting routines. One established fitting routine for “white-box” approaches is “lmfit”^13^ developed by Newville and contributors. As the detected spectra such as resonant inelastic X-ray scattering (RIXS),^5^ X-ray magnetic circular dichroism (XMCD),^14^ and time-resolved spectroscopies^15^ become more complex, users often have to create their own such “white-box” solutions and spend a significant amount of time developing and testing their fitting routines.^10^

Our previous challenges with fitting 2p3d RIXS spectra^1,4,5^ have led us to aim to integrate both approaches. Our goal is to create a user-friendly tool for fitting spectroscopic data. Furthermore, our fitting tool offers an uncomplicated approach to customizable methodologies. The proposed software tool, named SpectraFit, has been released as an open-source project on GitHub (https://github.com/anselmoo/spectrafit). Alongside the source code, we also provide documentation (https://anselmoo.github.io/spectrafit) that lists the program’s features and provides practical examples, which are cross-linked to the source code. We hope that this will provide our two target user groups (spectroscopists and computational chemists) with a deeper understanding of how each example works and how it can be customized to fit their specific needs. We believe that this approach further improves the transparency, shareability, and overall user-friendliness of the program because “Documentation is Like Code”.^16^

The SpectraFit program offers a comprehensive approach to data fitting, using both black-box and white-box techniques. The black box approach uses pre-existing fitting and data processing routines that are consistently integrated into the program. In contrast, the white box approach enables users to bind to a Jupyter-Notebook^17^ or import the program as a package in a different application, providing extensive customization options for data pre- and postprocessing (SI, Jupyter). With this approach, chemists are empowered to explore the interaction between data, model, and result, functioning as data engineers. The customized program is, in principle, able to handle all types of imported and exported data formats, but the fitting procedure of SpectraFit itself remains a core component that cannot be altered.

## Methods

### System Requirements

SpectraFit is a Python an open-source package licensed under the Berkeley Software Distribution (BSD) 3-Clause License^18^ that can be installed on Windows, Linux, and MacOS using pip or conda. It is compatible with Python versions 3.8 and higher. To ensure software quality, the unit tests are performed Pytest^19^ with a code coverage rate of 100%. The technical documentation is derived from the source code by following the Google Python Style Guide^16,20^ and is hosted on GitHub Pages. The software is standardized using Docker containerization technology, making it safe and accessible for interactive use (Jupyter-Notebook)^21^ and software development (Visual Studio Code (VSCode) Dev Containers).^22^

### Software Architecture

SpectraFit is an object-oriented design that is capable of catering to the needs of different users. It offers two distinct working modes, namely, the Command Line Interface (CLI) and Jupyter. CLI is designed to deliver rapid first results, while Jupyter is more focused on data engineering. Both modes require user input and input data to be provided separately:

1. In CLI mode, the user input is entered through the terminal or a user input file, while the input data is in the tabular form of a text file, such as a comma-separated file (CSV) with a column for energy and at least one column for intensity.
2. In Jupyter mode, the user input is passed as function values into the corresponding function ≪solver_model≫. Spectra data is directly imported as a Python Data Analysis Library (Pandas)^23^ DataFrame into the SpectraFit class ≪SpectraFitNotebook≫. This offers a high degree of user-specific freedom, forming the basis for data engineering with spectra.

**Figure 1:**
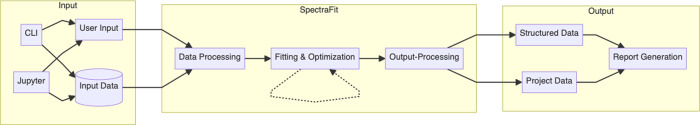
Schematic sketch of SpectraFit’s data workflow from input to output. By using the CLI or Jupyter Notebook, the user input and input data are selected. Next, data is passed to the core routine consisting of three subroutines: data processing, fitting and optimization, and output processing. Data processing refers to transforming the input data-based user input into a standardized machine-readable format.^26,27^ The input data will be analyzed and fitted using a standardized internal format, which is an iterative process. Once the fitting optimization is complete, the output processing or postprocessing is automatically performed, and a report can be finally generated. SpectraFit uses object-oriented software design to ensure that the modular, reusable software components, called objects,^28^ interact independently with each other throughout well-defined interfaces.

After importing data, the internally used data format (as shown in Figure 1, Block SpectraFit) remains consistent and relies on structured (Pandas) and unstructured (Python’s built-in dictionary) data types. The imported spectral data are handled in a tabulated form and attached to our fit data later. However, in order to meet project requirements and user needs, a more flexible approach is sometimes necessary. Therefore, metadata (Project Data, Figure 1) is stored in a dictionary and referred to as unstructured data. This means that elements such as types, length, used metric, and form can be set up individually. While it is possible for some parts of the unstructured data to contain structured elements, it is generally treated as unstructured data for simplicity.

The decision to use Pandas was influenced by its capacity to combine the numerical performance of NumPy^24^ with the versatile data handling and manipulation capabilities found in spreadsheet (relational) data designs. Pandas’ flexibility allows for easy storage and loading of data, as well as providing descriptive statistics and correlation analysis with just one library. In order to ensure that the data handling is consistent and accurate at all stages, we utilize the Pydantic^25^ data validation library, as shown in the workflow in Figure 1. Pydantic is used to validate the input data and transform it into the appropriate internal data format and then once again to validate the output data and convert it into the desired output format, complete with the correct data type and labels.

Version v1.0.0 of SpectraFit is developed with the support of the generative AI tools^29^ GitHub Copilot^30^ for code suggestions and cleaning and Sourcery^31^ for their specific code refactoring rules. Code refactoring does not affect code behavior; however, it is an integral part of software development to guarantee maintainability, readability, and code quality.^16,28^

## Results and Discussion

### Features and Usage

**Figure 2:**
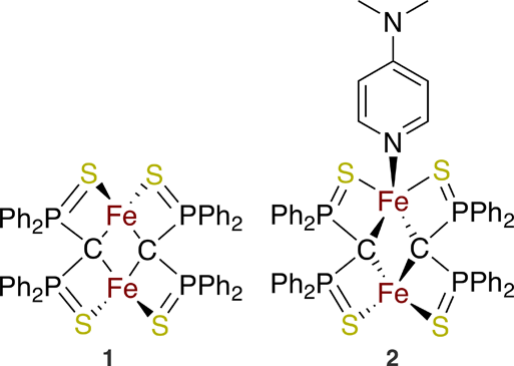
Chemical structure of complexes **1** and **2**.^7^ Adapted with permission from ref (7). Copyright 2023 American Chemical Society.

To demonstrate SpectraFit’s features and workflow, previously published X-ray spectra^7^ on iron–sulfur model complexes for nitrogenase are analyzed in this section; see also the “Notice to the Reader” at the end of the publication. Figure 2 displays the two complexes for this demonstration, two di-iron clusters [L_2_Fe_2_] and [L_2_Fe_2_]{DMAP}, labeled as **1** and **2**. Both complexes **1** and **2** have ligand (L = {[Ph_2_P(S)]_2_C}^2–^), while complex **2** is additionally substituted by DMAP (DMAP = [4-dimethylpyridine]). Complex **1** is an all-ferrous dimer 2Fe^II^, while complex **2** has mixed-valence Fe^II^Fe^III^.^7^ High-energy resolution fluorescence-detected X-ray absorption spectroscopy (HERFD XAS) was used to measure both complexes **1** and **2**, and these two XAS spectra form the basis for introducing SpectraFit.

Our aim is to showcase the abilities and potential of SpectraFit, a powerful tool for analyzing and interpreting spectroscopic data, by using this set of iron complexes. First, we will explain its basic features using the Command Line Interface (CLI). Second, we will provide an advanced walkthrough of a Jupyter Notebook example. Although we will use the same data set for both examples, the Jupyter Notebook will include additional features such as simultaneous fitting, statistics, and reporting capabilities, making it more complex. The gradual progress from simplicity (CLI) to complexity (Jupyter) illustrates characteristics and potential.

### Basic Features via Command Line Interface (CLI)

**Figure 3:**
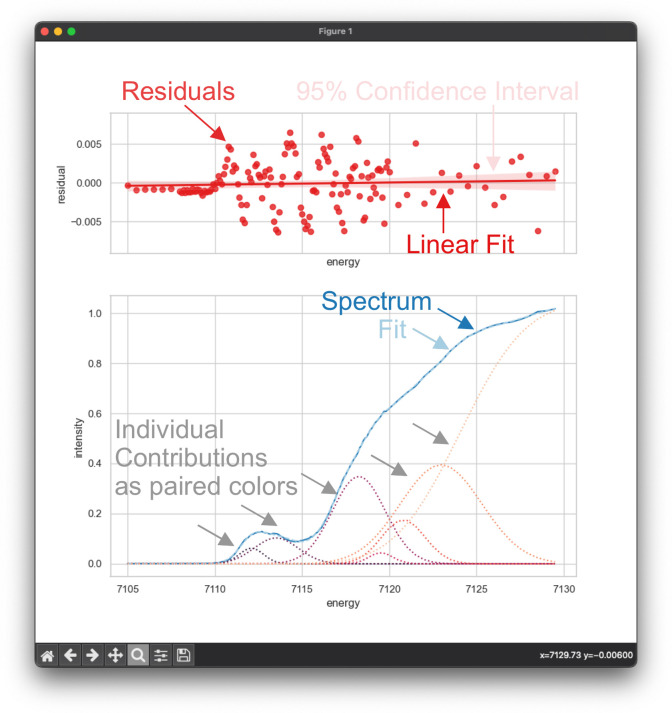
CMD plotting window via matplotlib^35^ showing a fit of the Fe K-edge XAS of the Fe^II^_2_S_2_-dimer (complex **1**). On the upper subplot, the residual (red dots) indicate the numerical variance between the raw data and the fit mode. A linear regression of the residual (red line) is also shown, together with the corresponding 95% confidence interval, which is generated by Seaborn’s regplot methodology.^36^ The regression plot is intended to help the user, the global accuracy of the fit to quickly identify areas of optimization. In this particular scenario, the straight line has a positive increase from 7105 to 7130 eV, which implies that the mean of residual is not zero and dominant and oscillating structure is visible in the area of pre- and rising-edge (∼7110–7120 eV). On the lower plot, the raw data is depicted by a blue line, while the fit model is represented by a dark blue dashed line. Dotted colored lines ranging from dark to light red are shown to represent individual contributions. The colors used in this visualization are generated by Seaborn’s rocket color palette.^36^ Adapted with permission from ref (7). Copyright 2023 American Chemical Society.

The SpectraFit tool, a robust data analysis and manipulation tool, offers two modes of operation: CLI and Jupyter Notebook. The CLI mode is best suited for quick, convenient fitting of individual spectra, like that of the all-ferrous complex **1** shown in Figure 3. In this operation mode, the syntax that needs to be used is “spectrafit ≪data.file≫ -i ≪input.file≫”. For more complex data analysis and seamless integration with other Python packages such as SciPy,^32^ the Jupyter-Notebook mode is recommended. The Advanced example section delves further into the Jupyter-Notebook mode. To generate the Residual (top) and Fit (bottom) spectra, as depicted in Figure 3, the user must input a textual format,^33^ such as JSON, YAML, or toml. The chosen format will then be translated into a built-in datatype dictionary, which represents an unordered collection of key-value pairs.^34^

**Figure 4:**
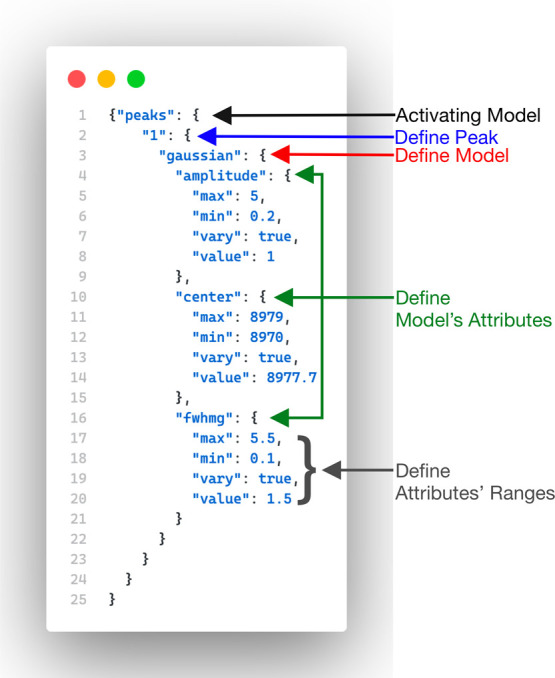
To define a distribution model, five attributes are required in an object-type format like JSON. The “peaks” attribute (black) initializes the distribution models, followed by its index (blue) as a string integer “1” for the first model. The first model is a Gaussian model (red) with attributes (green) such as amplitude, center, and full-width half-maximum for Gaussian (fwhmg). Finally, the minimum range (grew) (min), maximum range (max), verifying allowed (vary), and start value (value) complete the definition. It is important to note that only the distribution attributes that need optimization require definition; otherwise, default values are used, such as ≪Gaussian(x, amplitude = 1.0, center = 0.0, fwhmg = 1.0)≫.

The input data can be provided directly or as the absolute path in the input file. As shown in the input file (input.json) and the code snippets shown in Figure 4 (generated via CodeSnap^37^), there are two major objects within the input file:

1. Settings: With the settings block, cmd inputs can be overwritten, which can be helpful if the user wants to run the exact fitting multiple times with different filters, like shifting the energy axis or changing the fitting range.
2. Fitting: With the fitting block, the project description, lmfit-parameters (settings), and the model components can be defined. For the definition, a subdictionary has to look like in Figure 4:

This distribution model as a subdictionary is passed internally to the fitting function for each defined distribution model. A detailed description of all models is also provided in the Application Programming Interface (API) of the online documentation.

One important feature of SpectraFit to know is the support of lmfit’s^13^ string expression for distribution models. Rather than setting specific ranges and initial values for distribution models, the lmfit-solver can be instructed through *user-supplied expression*(38) to apply constraints to the fitting model. This feature is important for cases in which the underlying physics of the system should impose a constraint on the model, such as maintenance of a constant branching ratio^39^ for the amplitude (intensity) of L3- and L2-peaks when fitting L-edge XAS.^40^ Another scenario where using an automatized *user-supplied expression* is beneficial is during simultaneous fitting routines, where the peak energies between multiple spectra must remain constant. SpectraFit’s simultaneous fitting routine uses the initial user input to generate shared fitting parameters^41^ through internally autogenerated expressions. These expressions ensure that each spectrum is fitted with the appropriate set of distributions. By using the expression, the peak intensity (amplitude) and broadening can be confirmed, and the position (energy) value is frozen based on the expression’s internal dependency. Previously published results^4,42^ demonstrated the need for this routine, for example, for fitting RIXS, time-resolved UV–visible absorption measurements, or characterizing enzyme kinetics. For this study, complexes **1** and **2** illustrate how this routine works, following previously published methods.

After the fitting, graphical output is provided via the matplotlib package^35^ and shows the spectrum, its fit plus their individual components, and the corresponding residuals, as shown in Figure 3 above.

Python has a feature that allows a function to receive multiple keyword arguments with their corresponding values. This enables SpectraFit to use all the advanced fitting options and methods provided by the lmfit package^13^ in Python in a user-friendly and intuitive manner. For Figure 3, the “least_squares” minimization was performed the Trust Region Reflective method was chosen, which has proven to be highly efficient and robust method in solving nonlinear problems,^43^ and we highly recommend this method for your scientific requirements. However, the lmfit package^13^ offers 23 different fitting methods, as shown in the technical documentation,^44^ and the key-word-argument syntax of SpectraFit fully supports them.

**Figure 5:**
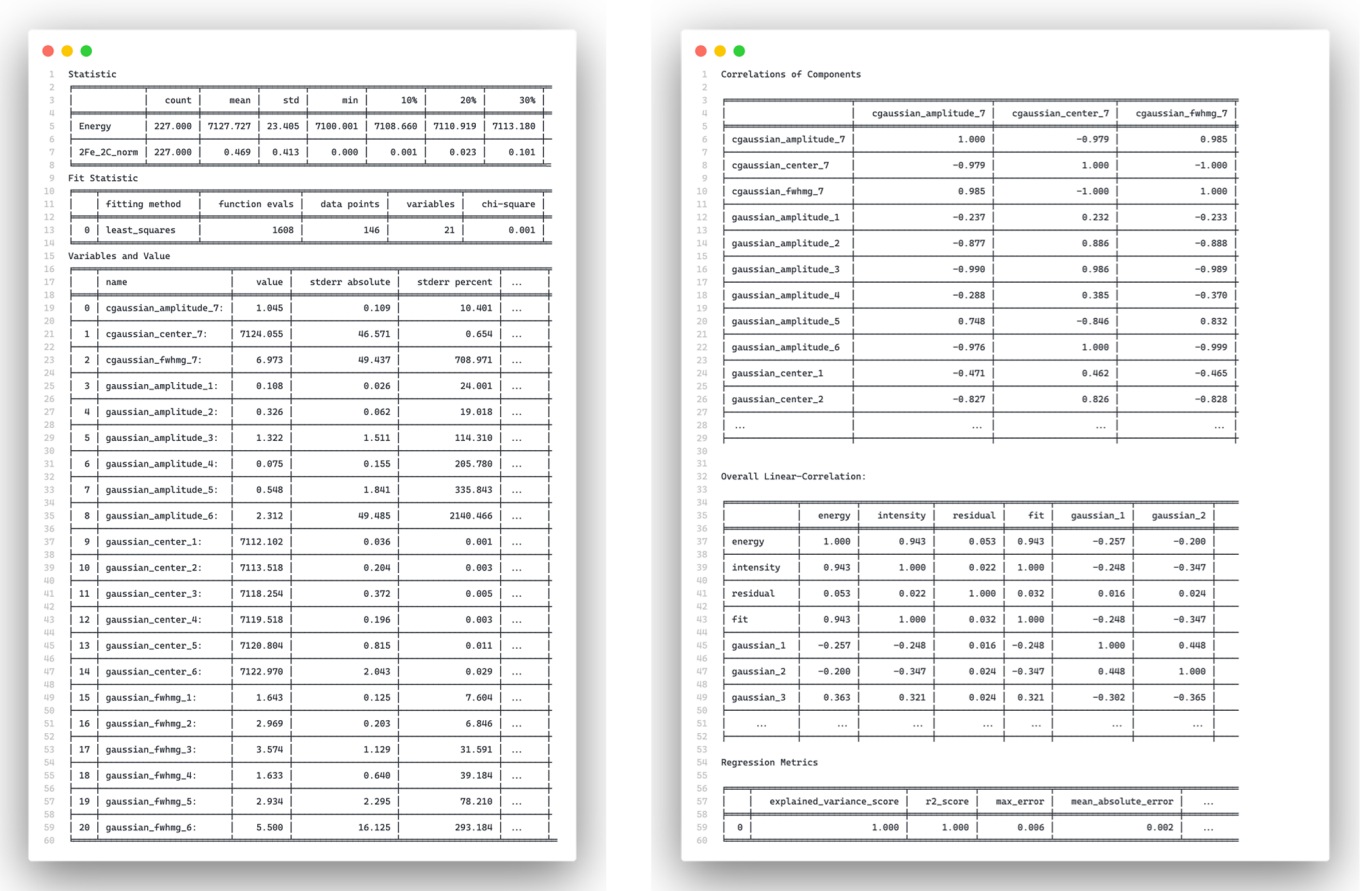
Capture of the default printout in the CLI before closing the program with its six default tables for evaluating the fit quality via descriptive^46^ “Statistics”, “Fit Statistics”, “Variables and Values”, the “Correlation of Component”, the “Overall Linear-Correlation”,^47^ and “Regression Metrics” analysis;^48^ the complete printout is provided as Figure S1. The CLI print feature in SpectraFit is designed to provide users with basic statistics about their input data set and fit results. If desired, this feature can be turned off, and all information will be automatically saved in the output file. An overview of the input XAS data for complex **1** (2Fe_2C_norm) that requires fitting is given in the initial section through descriptive statistics, including minimum and maximum values, the number of points, and additional information as outlined in the Built-in Statistics section. The initial and best values, as well as relative and absolute uncertainties (“stderror”) if available, are presented in the Variables and Values section. Additionally, in the case of optionally calculated confidence intervals, a table can also be presented if the calculation converges. Correlations between the different components of the model and variables like “energy” and “intensity” can be investigated via the component and overall correlations, which are also available in Table S1. The final table displays the details regarding the computed regression metrics. It is part of SpectraFit’s Data Exploration^49^ strategy to provide default statistical measurements to assist users in gaining confidence in the data and avoiding overinterpretation. Ultimately, data exploring and wrangling^50^ a critical to identifying patterns within the data itself.

Our findings indicate that the optimal models for fitting comprise of two peaks that are linked to the pre-edge, followed by three peaks that are associated with charge transfer and the rising edge regime.^7^ Our approach also uses cumulative Gaussian functions to represent the edge jump, which is associated with the ionization potential.^45^ Following each fit, we present descriptive statistics, correlation, and regression metrics in the CLI (refer to Figure 3) and provide a detailed analysis in the Builtin statistic section (Figure 5).

Once the plotting window is closed, the user will be prompted by the CLI to either continue or stop the fitting procedure, which automatically saves the results as CSV and JSON files. The CSV files consist of the fit, error analysis, and correlation analysis of the fit. On the other hand, the JSON file includes the entire fitting project, including user input, fit statistics, and fit results, as a user project.

In summary, the basic mode of SpectraFit should allow the user to fit spectra with a minimum of effort and to get a quick overview of the fit results. One of the main focuses is to provide the user with a tool that is easy to use and understand because the complexity of the underlying fitting package is hidden from the user. Therefore, the user input is kept as simple as possible, and the output is provided in a graphical and structured way. As part of the support of its simple philosophy, the following distribution models are delivered by default: Lorentzian, Gaussian, Pseudo-Voigt, Voigt, exponential function, step functions, and cumulative functions.

For users who require more advanced tasks, SpectraFit offers the option to access its implemented modules and create customized fitting procedures using a Python interface like the Jupyter Notebook. This is described in the Jupyter Notebook Support section below.

### Built-in Statistics

**Figure 6:**
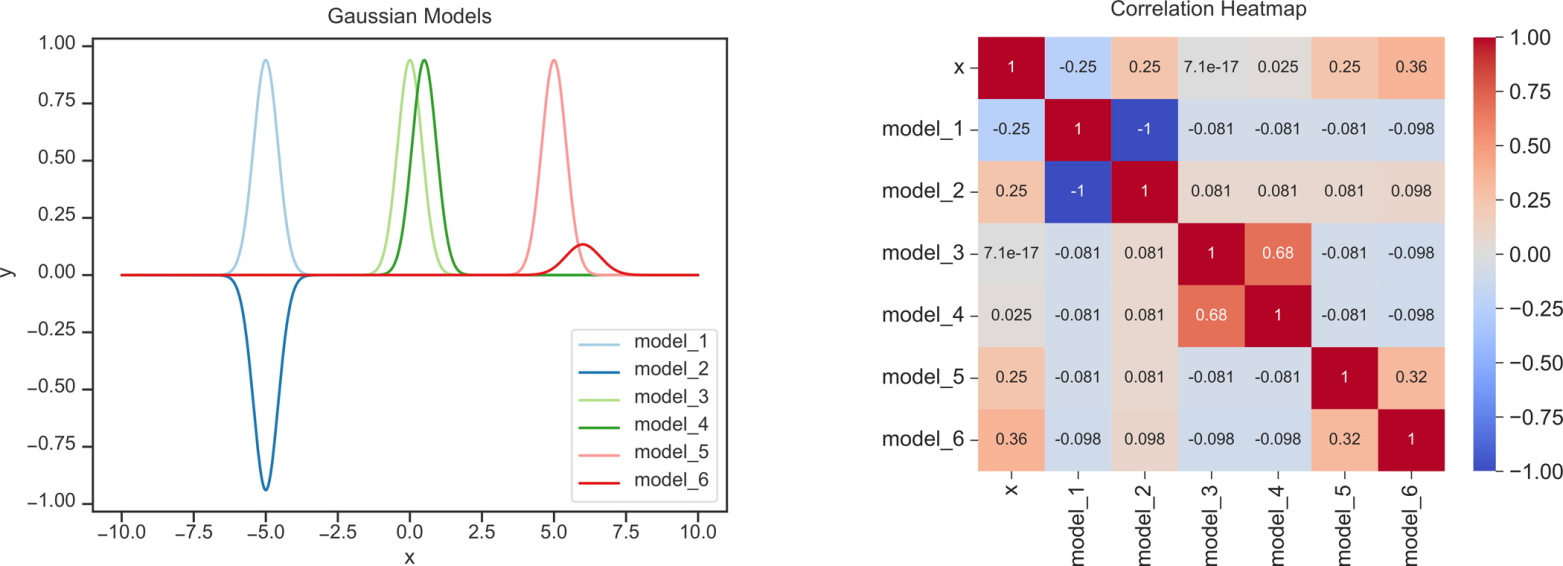
Graph (left) shows a model including six different arbitrary Gaussian functions. The heatmap (right) shows the corresponding linear correlation matrix. For example, the blue pair of Gaussians, “model_1” and “model_2” are identical functions with amplitude of opposite sign. This is reflected in their correlation coefficient of −1, which is represented by the blue color in the heatmap. Similarly, “model_3” and “model_4” have the same amplitudes and broadening but differ in center-shift by +0.5 on the *x*-axis. Thus, they are highly correlated, but only with a coefficient of +0.68, seen in the nearly red off-diagonal elements. Finally, the Gaussians “model_5” and “model_6” are shifted to each other with the variation in their amplitudes, resulting in a lower inner correlation of only 0.32.

**Figure 7:**
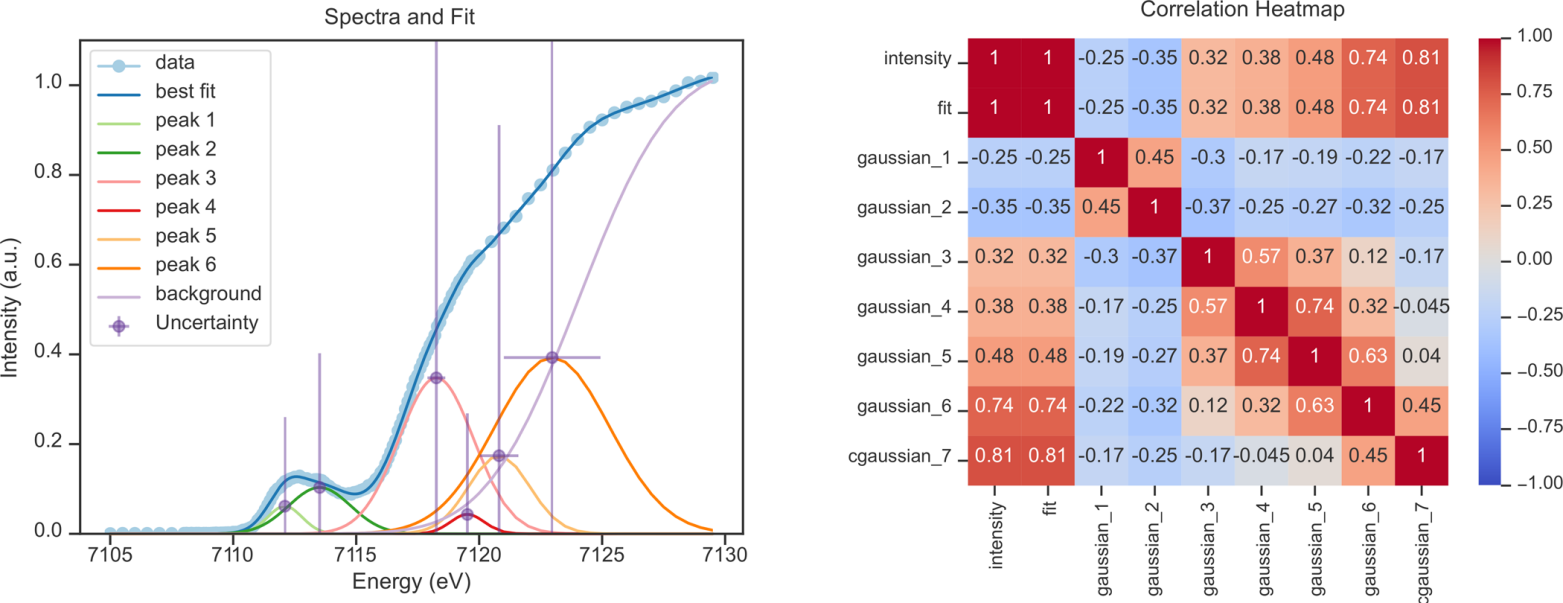
Normalized HERFD-detected Fe K-edge XAS spectrum of complex **1** (labeled “data”) has been fit to a model (left) including six peaks, represented by Gaussians, and a rising edge, represented by a cumulative Gaussian (labeled “background”). The plot (left) also shows the uncertainties of the amplitude and center variables of each component, while the corresponding correlation matrix is shown on the right. Adapted with permission from ref (7). Copyright 2023 American Chemical Society.

In addition to the built-in goodness of fit statistic^47^ in lmfit, SpectraFit also allows you to calculate the following statistics:

1. Descriptive statistics^46,50^ of the initial data are calculated by the Pandas package. This includes the mean, standard deviation, minimum, maximum, and 10% steps. This information helps get an overview of the data and check if the data is suitable for the fitting. Especially the trio of count, minimum, and maximum already gives the user a good idea about the fitting complexity.
2. The correlation analysis^47^ of the output data consists of the input spectrum, the fit, the residuals, and the single contributions; see also Table S1. Pearson-type correlation analysis is performed by the Pandas package.^23,51^ Based on the correlation analysis, the user can check whether the fit is reasonable and whether individual contributions are correlated with each other. The concept of correlation analysis is illustrated with an arbitrary model and its corresponding correlation matrix, shown in Figure 6, and an example using experimental data from complex **1** is shown in Figure 7. More generally, the correlation matrix, as shown in Figures 6 and 7, visually displays the linear correlation between model components, aiding in identifying possible simplifications; see also Table S1. The underlying lmfit package calculates BIC and AIC based on implemented fit statistics such as Chi-Square (χ2) and reduced χ2 (rχ2) analysis.
3. When using either the Levenberg–Marquardt (least) or least-squares minimization with the trust region reflective method (least_squares), SpectraFit attempts to calculate via lmfit^13^ the covariance matrices for both relative and absolute uncertainty by default. This measure of uncertainty in the energy and amplitude of individual components of a fit can be visualized as uncertainty bars, which can be generated using SpectraFit’s autoexport feature. In an unconstrained model, an optional confidence estimation can also be performed. The derivation of these uncertainty figures from the covariance matrix, alongside further details about the requirements and limitations of this feature, can be found in the SI section Uncertainties and Confidence Interval.

From the correlation matrix (Figure 7 (right)), two important observations can be made:

1. The linear correlation between the experimental intensity and the fit is 100%, which is ideal.
2. Gaussians 4, 5, and 6, in conjunction with the cumulative Gaussian, exhibit a correlation of over 50%, marking them as possible variables for model simplification. This may be taken into account for further efforts to optimize the fit statistics.

Uncertainty bars also (Figure 7 (left)) provide valuable information: the energies of the features in the pre-edge region are well-situated; however, the energy of all features that overlap with the edge-jump have significantly greater uncertainty. This is because these features are highly correlated to the cumulative Gaussian representing the edge-jump. This leads to a propagation of uncertainty from the cumulative Gaussian. It is well-known that the precise position of the edge jump is hard to define, so this is reflected in its overlapping features. Furthermore, the intensities of the highly correlated features also have large uncertainty bars. This is also true of the pre-edge region, which is resolved from the edge-jump but nevertheless consists of two strongly overlapping components. Therefore, the total intensity of peaks 1 + 2 is well-defined, but the exact intensity of each component has large uncertainty. It should be noted that the breadth of the cumulative Gaussian in the fit shown in Figure 7 is constrained. A fit of the same data set to a fully relaxed (i.e., unconstrained) model is shown in Figure S3. The unconstrained model allows for a calculation of confidence intervals, from 1σ to 3σ, also presented in Figure S4. The relaxed fit results in smaller uncertainty associated with the strongly correlated peaks but may not provide a physically reasonable picture of the situation. The balance between these considerations is a topic of great importance in quantitative applications of XAS, such as calculation of exact covalency^52^ or differential orbital covalency,^53^ and we hope that SpectraFit will be a valuable tool in improving the accuracy of such analyses.

4 SpectraFit’s regression metrics^48^ are implemented via the SciKit-Learn^54^ package, providing spectroscopists with a useful tool to analyze their model’s statistics; see also SI section Post-Processing via Scikit-Learn. Next to *classical* metrics like the mean absolute error (MAE), the mean squared error (MSE), and the root mean squared error (RMSE),^27,55^ SpectraFit also provides used *R*^2^ score^55^ and explained variance score. The *R*^2^, also known as the coefficient of determination,^47^ score is a statistical measure of how close the data are to the fitted regression line.^56^ It is also known as the coefficient of determination or the coefficient of multiple determination for multiple regression.^57^ The best possible score for *R*^2^ would be 1.0, and vice versa, the worst would be 0.0. In the case of a value of *R*^2^ = 1.0, the fit model explains all the variability of the dependent data set (spectrum).^47,57,58^ The explained variance score (EVS) is a regression score function and works similarly to the *R*^2^ score.^59,60^ Again, the best possible score is 1.0. It is important to note that the EVS relies on biased variance, whereas *R*^2^ uses raw sums of squares.^59,60^ There is ongoing discussion^59^ about using the EVS score or *R*^2^; however, the maintainer of Scikit–Learn recommends using *R*^2^ over EVS, because *R*^2^ takes a systematic offset in the prediction into account.^61^

### Jupyter Notebook Support

For interactive work, as recently became popular in chemistry,^62^ SpectraFit can also run in a Jupyter Notebook^17^ environment and uses the Plotly^63^ library for graphical presentation of the spectra.

**Figure 8:**
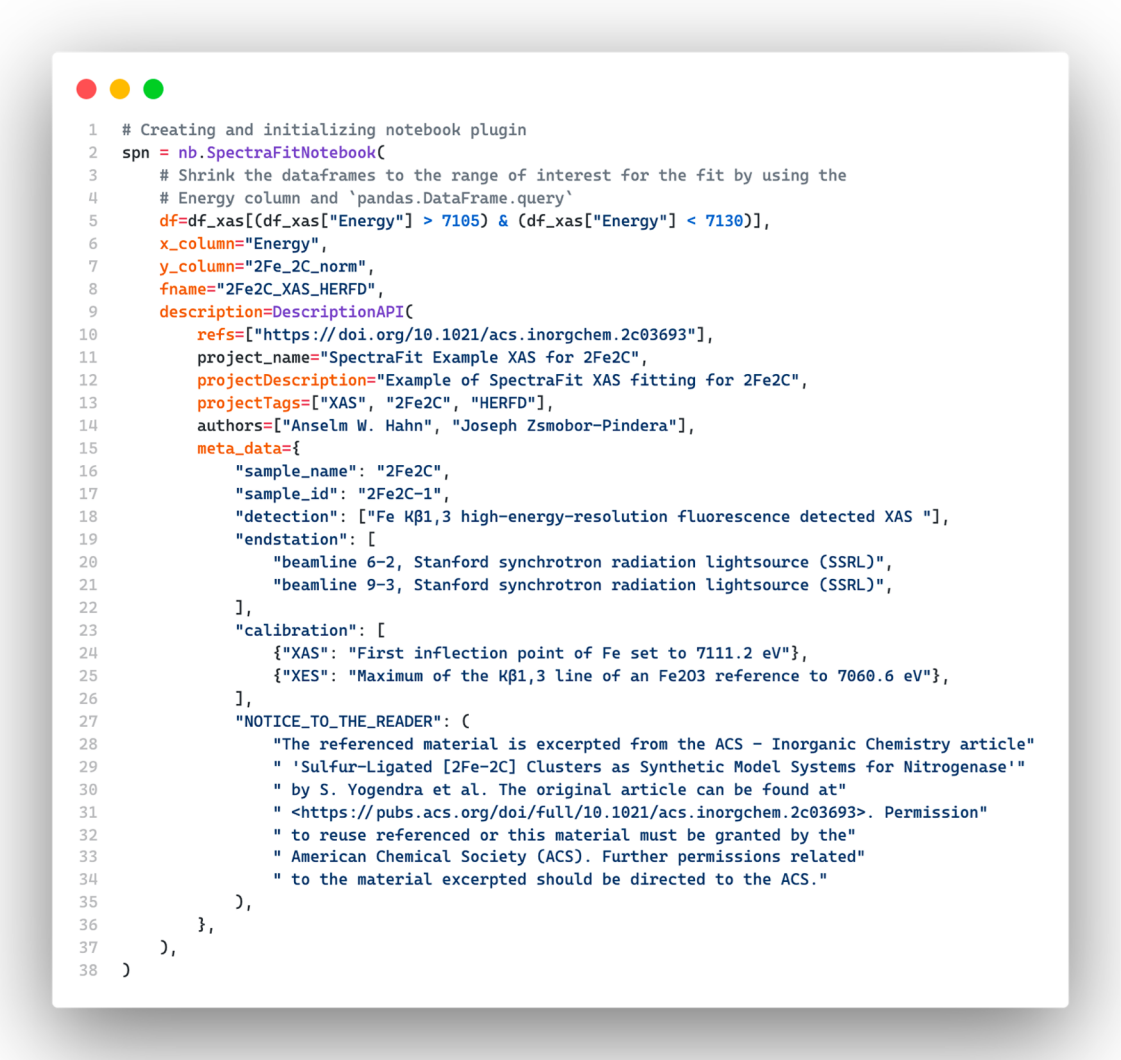
In this Python code snippet for a Jupyter Notebook cell, the imported SpectraFitNotebook object is used to fit HERFD-XAS data of complex **1** to a fitting model.

To use SpectraFit in Notebook mode for analyzing X-ray absorption spectroscopy (XAS) data, the “SpectraFitNotebook” class (Figure 8) has to first be imported. This class is responsible for managing and analyzing XAS data, but before starting the analysis, the user needs to define several parameters. First, the user must specify the DataFrame “df” containing the XAS data. The pandas DataFrame should be filtered to include only those rows where the “Energy” value is between 7105 and 7130 eV. Then, the user needs to specify the column names “x_column” and “y_column” that contain the *x* and *y* data for the fit. In this case, the *x* data is located in the “Energy” column, while the *y* data is in the “2Fe_2C_norm” column. Lastly, if desired, the user can redefine the parent name using “fname”. After defining these parameters, the XAS data can be analyzed using SpectraFit in Notebook mode.

To obtain a more detailed description of the fitting results, one can call an instance of the “DescriptionAPI” class. The description would include references, project name and description, tags, authors, and free-to-define metadata. In this particular case, the metadata would consist of details about the sample, detection method, endstation, calibration, and a message to the reader; see also page S26 of the SI.

Upon completion of initialization, the fitting model must be defined as a list of dictionaries; see Figures S10–S22.

**Figure 9:**
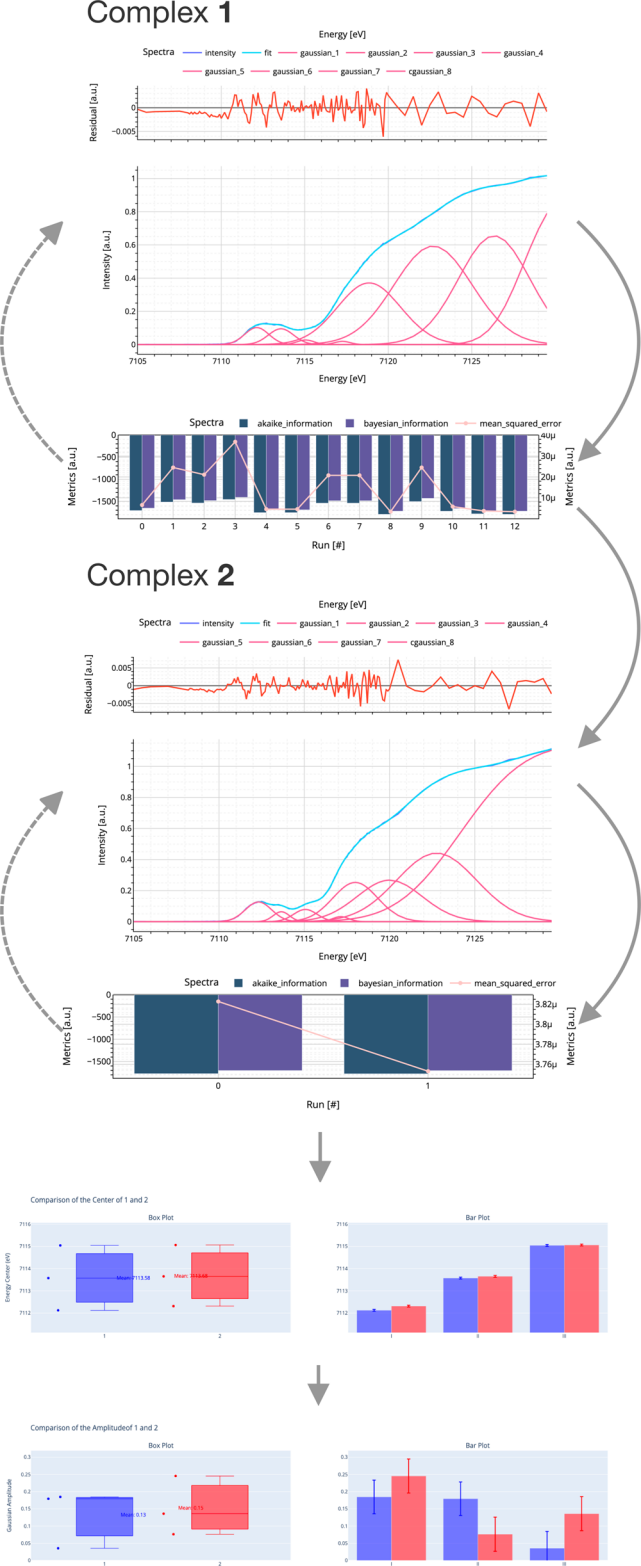
SpectraFit’s interactive Jupyter-Notebook interface is a powerful tool that allows users to explore the fit of complex **1** and **2**. The top of the interface displays the fit for the pre-, rising-edge, and K-edge, and users can interactively explore the spectrum, fit, and contributions using the Plotly^63^ feature. This feature includes built-in functions such as zooming, moving, and exporting, as well as mouse hover, making it easy to analyze the data. The bottom figure of the interface displays the fit statistics from cycle to cycle, including BIC and AIC as bars, and the MSE is displayed as an orange dotted line. In the bar graphs (bottom), a detailed comparison of three regions, labeled I, II, and III. This comparison shows a strong energy similarity and a slight amplitude difference. This level of detail is important for users who need to understand the nuances of their data and make informed decisions based on that understanding. In this particular context, the utilization of box plots to represent the amplitude and center of complexes **1** and **2** is a highly effective means of visualizing numerical data. The lower and higher extremes, as well as the interquartile range, are prominently displayed within these plots. Furthermore, the inclusion of a bar plot facilitates a direct comparison between the amplitude and center, with user-defined statistics employed for error estimation. Further details can be found in the SI, Jupyter. In essence, the user’s chosen amplitude statistic is computed by taking the standard deviation of each model and dividing it by the square root of three, which is the number of points. Meanwhile, the center statistic is determined by calculating the standard deviation of the difference between both complexes and dividing it by the square root of three as well. This process ensures accurate and precise measurements. The procedure to create these user-specific statistics is presented in Jupyter, SI, Advanced Section II. Adapted with permission from ref (7). Copyright 2023 American Chemical Society.

The graph shown in Figure 9 illustrates the best-fit solution for the normalized HERFD-XAS of complex 1 on the top. Although it may appear from the residual fits of complex 1 in Figures 3 and 9 that the Jupyter plugin provides more accurate results, this is not true. Both CLI and Jupyter use the same fitting backend, which implies that the data processing algorithms are hidden from the end user. Therefore, both approaches provide equivalent fitting outcomes (Figure S9).

One of the main differences between using Jupyter and CLI is the ability to interactively visualize graphics using Plotly,^63^ and view current numeric results as interactive tables via Pandas.^23^ With these interactive elements available in the Jupyter Notebook, the user can easily manipulate and explore the data. Thoroughly exploring data can lead to faster results by quickly eliminating unreliable models. The Jupyter Notebook can be useful for clarifying patterns in unfamiliar spectroscopic data.

**Figure 10:**
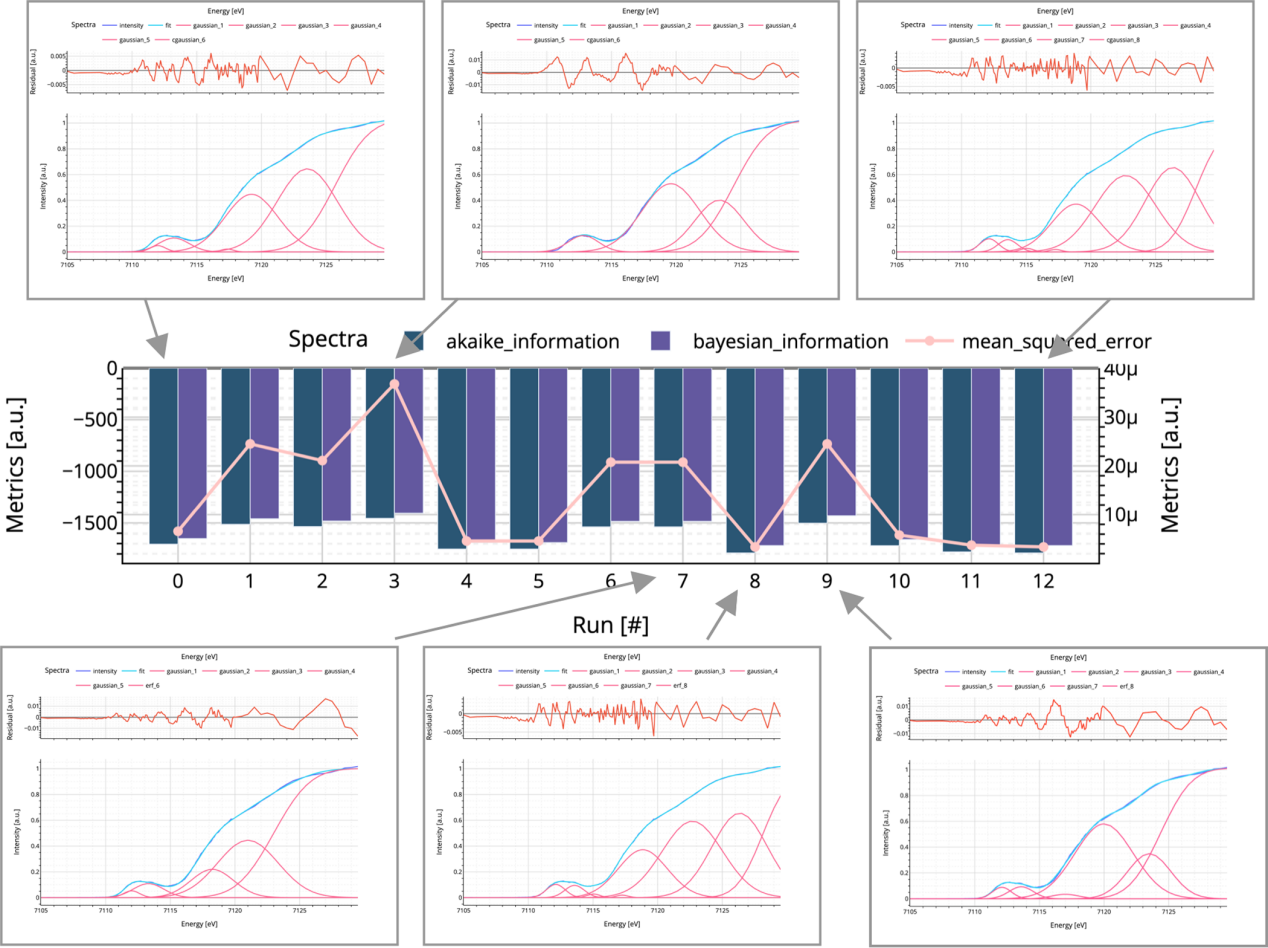
For following up on the development of the final fitting model of complex **1**, the corresponding metric is highlighted in the middle with its metric AIC (green), BIC (purple), and MSE (orange) for model validation. The corresponding plots for iteration steps 0 (initial guess model), 3, 7, 8, 9, and 12 (final model) are shown. The complete set of plots from 0 to 12 can be found in the SI (Figures S10–S22). At first, it may seem that all five fits represent the spectrum. However, the number of variables that best fits the data must be verified. Comparing the residuals of models 7 and 8 can help the user understand that both models might look equally good; however, the statistical metrics clearly indicate that model 8 is more accurate and reasonable. The statistical metrics plot shows that the addition of new variables to the model in the final model further improves the fit. This indicates that the presence of an additional feature in the edge region is more consistent with the data and serves as an example of the usefulness of SpectraFit’s statistical metrics plot. Adapted with permission from ref (7). Copyright 2023 American Chemical Society.

After iterative testing and validating various models (Figures 9 and 10), the final model can be conveniently stored by employing the class property generate_report, which exports a toml file with the suffix “*.lock” containing all information describing the fitting process of Figure 1. When it comes to testing and validating, the first step is to start with an initial guess model and optimize it until the fit metrics converge, as shown in Figure 10. By default, the fit metric includes AIC and BIC coefficients to show the causality of the model and MSE to highlight its accuracy. However, SpectraFit allows for individual configuration based on the implemented methods of goodness of fit statistics according to lmfit and regression metrics. By simply using one command, AIC and BIC can be replaced with rχ^2^ and MSE for the bar plot and *R*^2^ for MSE as a line plot, as highlighted in Figure S5. The metric criteria are handled as a list in SpectraFit, allowing them to be extended up to user-recommended specifications. An effective way to improve this optimization process for spectrum fitting is to incorporate Machine Learning concepts,^17,56,64^ starting hyperoptimization and ultimately including unsupervised learning. This can help mitigate the impact of user bias^9^ and automate the process.

Once the HERFD-XAS of complex **1** fitting is completed, the same input guess is utilized for the absorption spectra of complex **2** (Figure 9, middle plot). After fine-tuning, the results appear to be sufficiently accurate, precluding any further optimizations. Here, the primary benefit of Jupyter (Figure 9, bottom plots) is to provide the user with a playground to explore the data and also to tell insightful stories out of the data due to its didactic potential.^17,49,65^

Through the utilization of data-driven storytelling techniques, three dominant peaks in both spectra spanning a range from 7112 to 7115 eV have been successfully identified. All three peaks were found to be in close proximity to each other, with an energy difference of less than 0.19 eV through independent fits. Peaks I and II were associated with transitions from the 1s core hole to the 3d manifold,^7,45,66^ while peak III of complex **2** was previously thought to be a metal-to-metal charge-transfer (MMCT) transition.^7^ The Gaussian distribution used for the assumed MMCT transition can also be applied to the fitting model of complex **1**, which is interesting. In terms of amplitude comparison, a significantly more substantial variation was exhibited by mixed-valence complex **2** than complex **1**, although their mean values were similar to a difference of approximately 13%.

It should be noted that this study is not intended to overturn any existing trends but rather to provide valuable insight into how future X-ray studies of highly covalent complexes can benefit from analysis through SpectraFit. The next step involves the application of a global fitting model to both spectra to determine if the corresponding metric becomes even better (more causality and accuracy).

In summary, the Jupyter Notebook interface is an incredibly useful tool that allows for interactive changes to fitting parameters, immediate results, and a comprehensive analysis of all aspects of data analysis (code, analysis, and visualization) in one convenient location. Results can be tagged and compared for further analysis through the use of the versionization tool git.^67^

### Simultaneous Fitting with Shared and Constraint Parameters

Fitting multiple spectra accurately can be challenging as it requires simultaneous fitting with shared and constrained parameters. In magnetic circular dichroism (MCD) measurements, fitting all temperature-dependent spectra to one model with fixed energy but varying amplitude and broadening is an established approach,^4^ which is sometimes referred to as global fitting, although the term is not entirely accurate. SpectraFit supports the fitting of spectra with simultaneous fitting with shared and constrained parameters.

SpectraFit’s fitting routine allows the user to simultaneously activate this fitting mode for all required spectra, without the need to provide the fitting model for each spectrum separately.

As mentioned before, *global* fitting is important in chemistry^4,42^ because it allows for the simultaneous fitting of multiple data sets to a single model, which can improve the accuracy and reliability of the model parameters. It is also important for complex models with many parameters, as it can help to reduce the risk of overfitting^68^ and consequently misinterpreting. In combination with the goodness of fit statistics and correlation analysis, the user can statistically investigate the reasonability of the fit; in the SI, Advanced Usage III, global fitting is utilized to empirically showcase the effect of covalency^40^ on XAS spectra.

When analyzing complexes **1** and **2**, it has been observed that the peak energies for both pre- and rising-edge reasons tend to be similar, as shown in Figure 9. To further improve the BIC and AIC criteria while maintaining an acceptable MSE, it would make sense to test a simultaneous fitting model for both complexes together. However, before starting the fit, it should be noted that both spectra will be combined and then reshaped back into two again.

**Figure 11:**
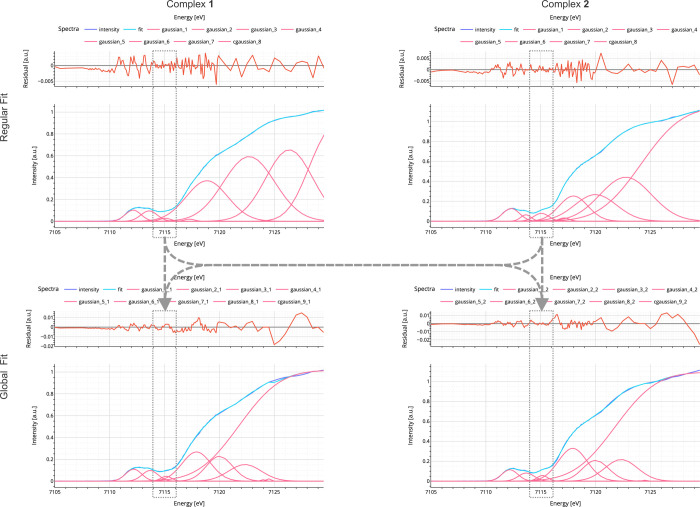
Above graphs display the fitting outcomes for complexes **1** and **2**, using separate models, while the graphs below illustrate the fitting results using a simultaneous model. The focus of the current observation is how peak III in all four spectra is described, which is indicated by the dashed boxes. All peaks are placed at the same energy position on both spectra for complexes **1** and **2** in the bottom plots. Upon closer inspection of the spectra and corresponding fit, it is evident that the model fit still matches both spectra with high accuracy. However, comparing the residuals shows a decrease in precision in the region beyond the edge jump. It is crucial to note that the improvement in causality measured by BIC and AIC is significant. Adapted with permission from ref (7). Copyright 2023 American Chemical Society.

As a result, the sample size will double from 2 × 146 to 292 and also the number of required variables for the distributions, as shown in Figure 11. Specifically, the simultaneous fitting model will require 36 variables or only 18 per spectrum, while the individual models will require 24 for complex **1** and 21 for complex **2**, respectively. It is worth noting that SpectraFit not only makes it easy to engineer the data, but it also applies the same philosophy to reports, making them easy to read via the toml schema and safe with a unique and unalterable ID for exploring the data; see also SI page S25.

With the effective variable reduction per spectrum, it is now interesting to see how AIC, BIC, and MSE behave when switching from individual fit solutions to one simultaneous fit solution. Table 1 shows that the MSE, χ^2^, and *r*χ^2^ increase by approximately one magnitude for complex **1**; however, the common AIC and BIC also improve up to a factor of 1.75.

**Table 1:** Assessment of Statistical Adequacy Necessitates Utilization of BIC, AIC, MSE, χ^2^, and *r*χ^2^ for Regular Fits and Simultaneous Fit of the Dimer Series^a^

	Regular fit of **1**	Regular fit of **2**	Simultaneous fit of **1**	Simultaneous fit of **2**
BIC	–1.7193e+03	–1.7044e+03	–2.9932e+03	
AIC	–1.7909e+03	–1.7760e+03	–3.1256e+03	
MSE	3.3875e–06	3.7531e–06	1.5963e–05	1.9130e–05
χ^2^	4.9457e–04	5.4795e–04	5.1236e–03	
*r*χ^2^	4.0539e–06	4.4914e–06	2.0014e–05	

a In reference to BIC and AIC, a more negative score indicates superior outcomes, while a lower value for MSE, χ^2^, and *r*χ^2^ signifies better performance. BIC and AIC are employed to appraise the global level of fitness of the model. Conversely, the regression metric is calculated for each spectrum (true) against its corresponding fit (predicted) on an individual basis.

After fitting the individual model-based versus single model-based results, the user faces the challenge of determining the more chemically reasonable model. With the simultaneous fitting model, the user has to consider that in the presence of strong covalency, the absolute energies may not be the best indicator of the electronic structure (Figure 11). This topic has been discussed in the literature.^7,45,66^ For complex **2** and its MMCT at 7115.2 eV, the peak with the same energy but different amplitude (**1**: 0.055 au vs **2**: 0.069 au → ∼20% increase in amplitude) is also an important piece to describe the spectra of complex **1** via a fitting model.

Finally, we want to emphasize how user-friendly (formally: ≪y_column = “2Fe_2C_norm”≫ or ≪“2Fe_2C_DMAP_norm”≫ , now: ≪y_column = [“2Fe_2C_norm”, “2Fe_2C_DMAP_norm”]≫) and safe it is to change from regular fit to simultaneous fit by just providing a list of spectra. Figure 11, along with the SI, Advanced Usage III, highlights this aspect. The Open-Source Community aspect welcomes feedback for further improvements and extensions.

## Conclusions

We present SpectraFit, a Python package specifically designed for the analysis of spectroscopic data using various fitting and statistical tools. This package’s object-oriented nature allows for the full utilization of the range of fitting optimizers and minimizers available in its underlying fitting packages. Additionally, SpectraFit offers a Python API, which allows the user to access the full potential of the fitting package. Our research demonstrates the effectiveness and efficiency of SpectraFit in analyzing spectroscopic data. Both a command line and a Jupyter interface with the graphical user interface (GUI) via Plotly^63^ are provided and allow to work on fitting problems in a project fashion, including standardized file export. Ultimately, the pledge for open-source development provides a platform for the community to extend the functionality of SpectraFit by suggesting new improvements, extensions, and plugins via the issue and pull requests.

## Supporting Information Available

Text file of processed XAS spectra for the referenced material,^7^ environmental settings, and high-resolution images (PDF)
README and input and output files for the CLI and Jupyter Notebook to replicate the results that were published (ZIP)

## Notes

The referenced material^7^ is excerpted from the ACS - Inorganic Chemistry article “Sulfur-Ligated [2Fe-2C] Clusters as Synthetic Model Systems for Nitrogenase” by S. Yogendra et al. The original article can be found at https://pubs.acs.org/doi/full/10.1021/acs.inorgchem.2c03693.

## Notes

Permission to reuse reference (7) or this material must be granted by the American Chemical Society (ACS). Further permissions related to the material excerpted should be directed to the ACS.

## Notes

The code snippet images for Figures 4, 5, and 8 were created using the CodeSnap^37^ extension in VSCode.
