# Supplementary material for: Introducing SpectraFit: An Open-Source Tool for Interactive Spectral Analysis
Source: ACS Omega. 2024 May 20;9(22):23252–65. doi: 10.1021/acsomega.3c09262 (PMC11155667; doi:10.1021/acsomega.3c09262)
Supplement: Supplementary file 1 — ao3c09262_si_001.pdf [file ao3c09262_si_001.pdf]

Supporting Information

Introducing SpectraFit: An Open-Source Tool for Interactive Spectral Analysis.

Anselm W. Hahn[1,*], Joseph Zsombor-Pindera[2,3], Pierre Kennepohl[2], and Serena DeBeer[1]

[1]Max Planck Institute for Chemical Energy Conversion, Stiftstraße 34-36, 45470 Mülheim an der Ruhr, Germany

[2]Department of Chemistry, University of Calgary, Calgary, AB, T2N 1N4, Canada

[3]Department of Chemistry, The University of British Columbia, Vancouver, BC, V6T 1Z1, Canada

Corresponding Author

*E-mail: anselm.hahn@gmail.com

Table of Contents

PROMPT OUTPUT 2
CORRELATION 4
UNCERTAINTIES AND CONFIDENCE INTERVAL 6
CORRESPONDING TABLES FOR FIGURES 10
CHANGING THE TYPE OF METRIC 25
ABOUT `DESCRIPTIONAPI` AND `*.LOCK FILES` 26
POST-PROCESSING VIA SCIKIT-LEARN 27
PLUGINS 30
CLI VS. JUPYTER 31
FIT SERIES OF FIGURE 10 FOR COMPLEX 1 32
REFERENCES 39

Prompt Output

The prompt outputs of Figure 2 and the relaxed model Figure S1 are presented in Figures S4 and S5, respectively. As mentioned earlier, there may be instances where it is not possible to obtain confidence intervals despite successfully calculating uncertainties. Hence, Figure S4 lacks confidence intervals. However, in the case of the proposed model of Figure S2, the corresponding table is highlighted in Figure S5.

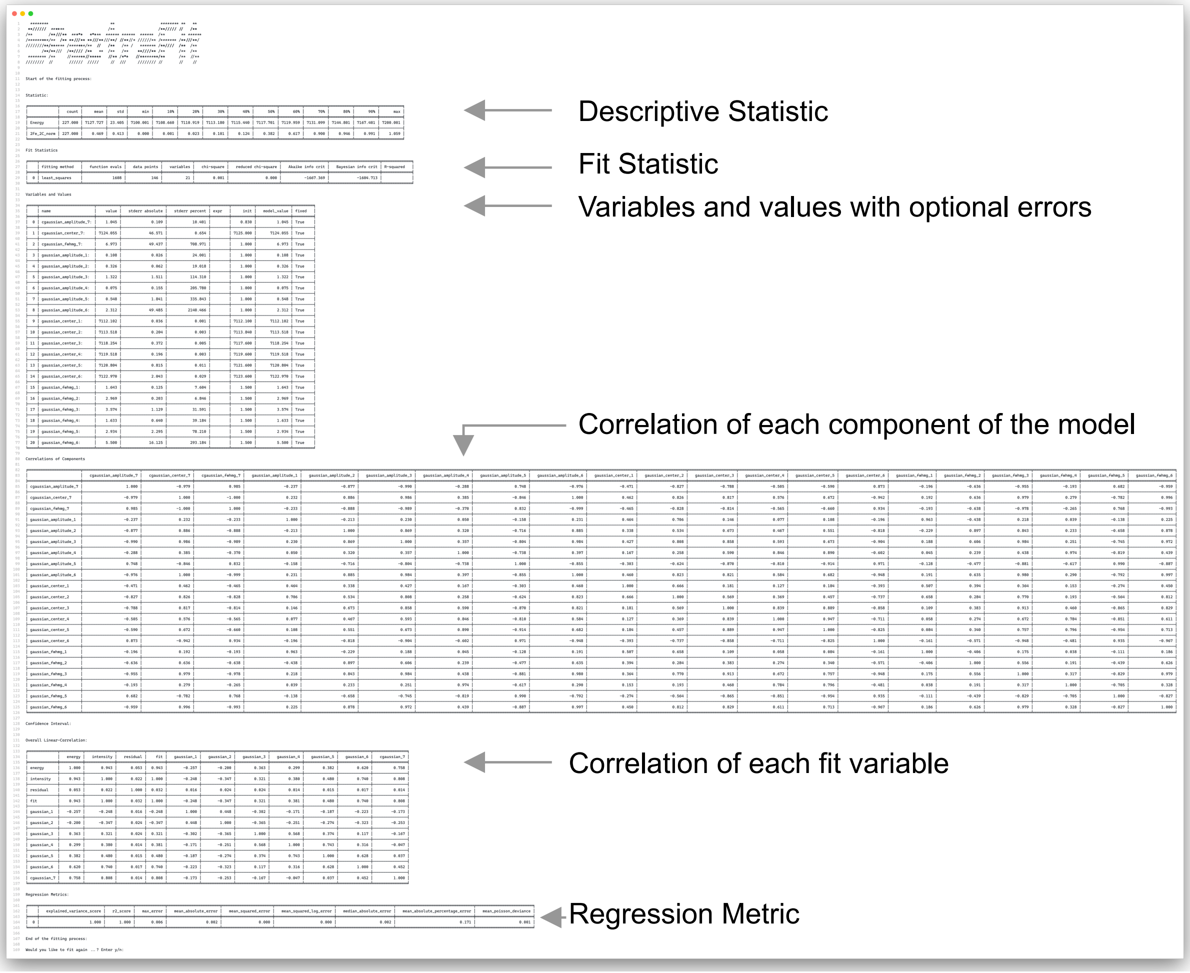

Figure S1. The complete CLI prompt of Figure 5, which is displayed in the user's terminal. The printout always includes six types of tables, such as descriptive statistics, fit statistics, variables with optional errors, correlation of each model component, general correlation of each fit variable, and the regression metric. Additionally, the confidence intervals can be provided as an optional table in the terminal for the user.

Confidence Interval:

	99.73% - LOWER	95.45% - LOWER	68.27% - LOWER	BEST	68.27% - UPPER	95.45% - UPPER	99.73% - UPPER
gaussian_amplitude_1	-0.02727	-0.01972	-0.01074	0.07421	0.01261	0.02697	0.04300
gaussian_center_1	-0.06599	-0.03706	-0.02595	7111.92722	0.02793	0.04165	0.09133
gaussian_fwhmg_1	-0.19492	-0.12952	-0.06456	1.35162	0.06454	0.12970	0.19695
gaussian_amplitude_2	-0.06205	-0.04090	-0.02042	0.22887	0.02105	0.04319	0.07482
gaussian_center_2	-0.11259	-0.08543	-0.06644	7113.10943	0.07194	0.10424	0.18539
gaussian_fwhmg_2	-0.36655	-0.23179	-0.11135	2.33468	0.10567	0.20703	0.33316
gaussian_amplitude_3	-0.05456	-0.05348	-0.01159	0.06095	0.01452	0.03400	0.06838
gaussian_center_3	-0.08722	-0.05059	-0.03460	7117.30005	0.04186	0.05831	0.11080
gaussian_fwhmg_3	-0.27560	-0.18244	-0.09092	1.34459	0.09198	0.18776	0.29997
gaussian_amplitude_4	-0.17672	-0.12523	-0.06564	0.26026	0.42991	0.42992	0.58613
gaussian_center_4	-0.39131	-0.20617	-0.11737	7118.71376	0.09001	0.13080	0.21780
gaussian_fwhmg_4	-0.73074	-0.45824	-0.22073	2.60351	0.21630	0.43422	0.86741
gaussian_amplitude_5	-0.90338	-0.54992	-0.20868	1.11824	0.23042	0.57612	inf
gaussian_center_5	-0.75156	-0.44856	-0.30544	7120.40297	0.40515	0.61497	1.41901
gaussian_fwhmg_5	-2.72076	-1.46219	-0.52089	5.32219	0.55143	1.18320	1.65864
gaussian_amplitude_6	-0.17488	-0.13318	-0.07703	0.23754	0.10847	0.26821	0.51548
gaussian_center_6	-0.86275	-0.46801	-0.33176	7124.50850	0.21531	0.35932	0.56750
gaussian_fwhmg_6	-1.04861	-0.69983	-0.35804	3.36652	0.39368	0.85862	1.44929
cgaussian_amplitude_7	-0.01597	-0.01125	-0.00601	1.04639	0.00730	0.01679	0.03022
cgaussian_center_7	-0.61836	-0.37298	-0.25868	7121.47739	0.14990	0.61831	inf
cgaussian_fwhmg_7	-inf	-0.65277	-0.26254	9.82044	0.23789	0.49032	0.78901

Figure S2. Terminal output as confidence table for the relaxed model of Figure S4.

Correlation:

The linear correlation is a helpful technique for SpectraFit users as it enables them to answer fundamental questions on a qualitative level, without needing to perform the fitting process repeatedly. Through analyzing the correlation matrix generated via the linear correlation technique, SpectraFit users can save time by avoiding trial and error and promptly obtaining a reasonable model.

The Pandas[2] library provides the linear correlation, which employs a statistical technique to measure the strength and direction of a linear relationship between two variables. This technique is particularly useful when dealing with tabulated data, as it helps to assess how closely the values of one variable correspond to those of another.

The formula for calculating the linear correlation coefficient (r) is as follows:

$$r = \frac{n(\sum xy) - (\sum x)(\sum y)}{\sqrt{[n\sum x^2 - (\sum x)^2][n\sum y^2 - (\sum y)^2]}}$$

Here, (n) denotes the number of data points, and (Σ) represents the sum of values. The linear correlation coefficient ranges from -1 to +1. A value (r) of +1 signifies a perfect positive linear relationship, -1 indicates a perfect negative linear relationship, and 0 implies no linear relationship.

In order to calculate the linear correlation, the fit results are used, which are stored internally as a Pandas DataFrame.[2] The following columns are used as 1D-Arrays:

1. Energy
2. Intensity (Spectrum)
3. Residual (difference between intensity and fit)
4. Fit (sum of all single optimized distributions)
5. Single optimized distributions (each as a single column)

These are automatically processed and converted into a correlation matrix, which is presented as a tabulated dataframe with $n\,x\,n$ columns. By analyzing this data qualitatively and answering a few basic questions, you can gain further insight into the data as provided in Table S1.

Table S1. Respond table about the correlation between variables and their importance.

Variable 1	Variable 2	Question
Energy	Intensity	Is there a connection between the changes in energy levels and the corresponding changes in the intensity of the spectrum?
Intensity	Fit or Residual	How strongly are the fit or corresponding residual intensity correlated with the spectrum intensity? Ideally, the correlation should be 1 for the fit and 0 for the residual.
Single optimized distributions - A	Single optimized distributions - B	Are there any correlations among the single optimized distributions? Exploring the relationships between these distributions can reveal underlying patterns in the spectral data, which can help to simplify or reduce the fitting model.
Intensity	Single optimized distributions	How do the individual optimized distributions correlate with the overall intensity of the spectrum? Insights into the overall spectral intensity can be gained by understanding the correlations and contributions of each distribution.
Energy	Single optimized distributions	Is there a correlation between energy levels and a particular single optimized distribution? This can help identify the energy regions where specific distributions are more prominent, such as edge jump or post-edge regions in XAS.

Finally, the user should monitor for any outliers in the correlations that may indicate unusual behavior in specific areas or conditions.

Uncertainties and Confidence Interval

SpectraFit attempts to calculate the covariance matrix by default for the Trusted Region algorithm provided by lmfit.[3] However, it is possible that the matrix could be empty, resulting in the inability to estimate uncertainty. This can occur if the model is overly constrained and during optimization, one or more parameters are limited to a boundary region. In such cases, even a single hit can result in an empty covariance matrix.
It is important to take into account the definition of the model when calculating uncertainties and displaying uncertainty bars. For Gaussian and Cumulative Gaussian models, the height is defined as the amplitude divided by the broadening. Therefore, both types of errors, amplitude and Full Width Half Maximum (FWHM), should be considered when calculating uncertainties using the equation:

$$h(e) = \frac{e_{\text{amplitude}}}{e_{\text{fwhm}} \cdot \sqrt{2\pi} \cdot \frac{1}{2\sqrt{2\ln(2)}}}$$

In order to understand uncertainties and confidence intervals in SpectraFit, it is important to start with the basics. Uncertainty refers to the amount of error or variance in an estimate,[4] while confidence intervals[5] provide a range of values within which the true value is likely to fall. To accurately estimate uncertainties and calculate confidence intervals, a model with no constraints is required. This type of model is referred to as a relaxed model by us, as shown in Figure S3. By using this model, we can obtain precise insights into the estimates and accurately calculate the confidence intervals.

When it comes to parameter estimation, the covariance matrix[4] is the tool for quantifying uncertainties associated with estimated parameters; currently, only Levenberg-Marquardt and Trusted Region algorithms are supported in SpectraFit. The process begins with estimating model parameters (θ). This process involves computing residuals (r), representing the differences between observed values (y) and model predictions ($\mathrm{f}(\mathrm{x}, \theta)$). The Jacobian matrix ($J$), derived from partial derivatives of residuals with respect to parameters, is crucial in capturing the sensitivity of residuals to parameter changes.
Next, the covariance matrix $\left(\mathrm{Cov}\left(\hat{\theta}\right)\right)$ is then calculated using the Jacobian matrix (J). This matrix provides a comprehensive view of uncertainties in parameter estimates. The diagonal elements indicate the variances of individual parameters, signifying the magnitude of uncertainties. Larger values suggest higher uncertainties (Figure S3). Meanwhile, the off-diagonal elements represent covariances between pairs of parameters, revealing the extent to which changes in one parameter correlate with changes in another.

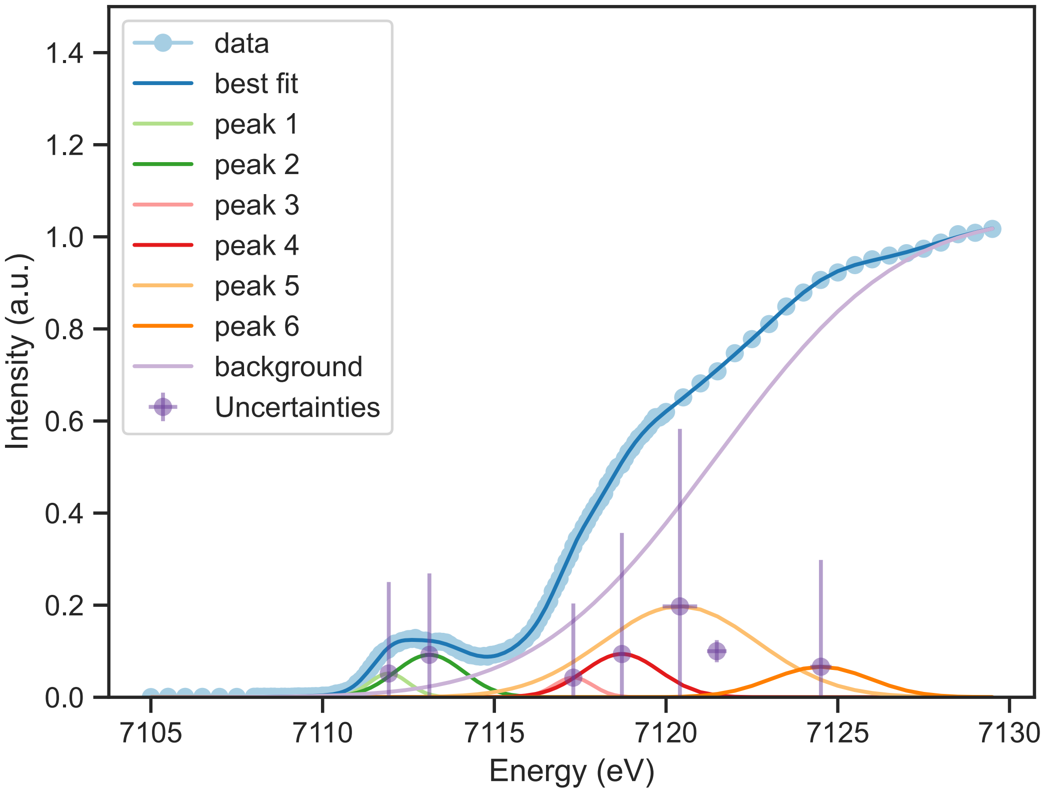

Figure S3. Relaxed fit with uncertainties bars. Adapted with permission from Ref. 1. Copyright 2023 American Chemical Society.

The formula for the covariance matrix involves the variance of residuals (σ^2) and the inverse of the squared Jacobian matrix ($(J^T \cdot J)^{-1}$). Standard errors $\left(SE(\hat{\theta}_i) = \sqrt{Var(\hat{\theta}_i)}\right)$, derived as the square roots of the diagonal elements, quantify the uncertainty associated with each parameter estimate. These standard errors become crucial in constructing confidence intervals for the parameters, providing a range within which true parameter values are likely to lie with a certain level of confidence.

It is important to know that the covariance matrix not only provides insights into the individual uncertainties of parameters but also captures the interdependencies between parameters, offering a comprehensive understanding of the uncertainties inherent in the parameter estimation process.

After getting the covariance matrix, the next step is to carry out an *F*-test.[6] This test measures the overall significance of the model by comparing the mean square of residuals to the mean square of the model. The *F*-test results guide the construction of confidence intervals.
For each parameter, the standard error is multiplied by the critical value from the *F*-distribution, yielding a margin of error according to $ME_i ct_{\alpha/2,df} \cdot SE(\hat{\theta}_i)$, where $t_{\alpha/2,df}$ t is the critical value from the *t*-distribution with $(n-p)$ degrees of freedom (n is the sample size, p is the number of parameters). This subset is added and subtracted from the parameter estimate, establishing the lower and upper bounds of the confidence interval, so that the confidence interval is finally defined as $\mathrm{CI}_i = [\hat{\theta}_i - ME_i, \hat{\theta}_i + ME_i]$. SpectraFit allows us to calculate this via limit; however, it is important to know for the reader that there is no guarantee to obtain

these values if the optimization does not converge. Furthermore, infinity values are possible (Table S2) if the standard error is already very small and the probing creates a zero division.

Expressing these intervals in terms of sigma levels provides an intuitive measure of confidence as shown in Figure S4. A 1σ-interval, corresponding to one standard deviation, conveys a 68.27% confidence level. Expanding to 2σ- and 3σ-intervals widen the range, offering 95.45% and 99.73% confidence levels, respectively.

When interpreting the results, wider intervals suggest higher uncertainties, while narrower intervals signal more precise parameter estimates. By integrating information from the covariance matrix and the insights derived from the F-test, it can be obtained confidence intervals that offer a robust and interpretable framework for understanding the uncertainties associated with least squares parameter estimates.

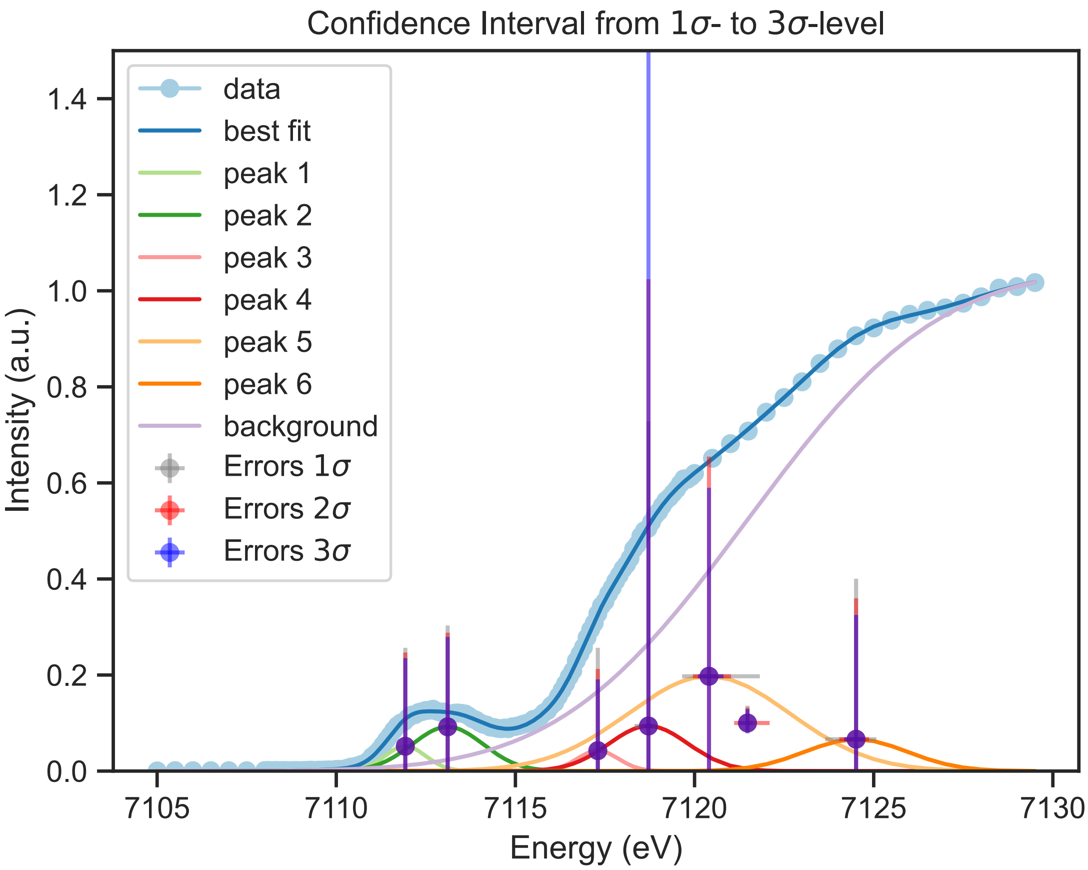

Figure S4. Relaxed fit with confidential integrals from 1- to 3-$\boldsymbol{\sigma}$. Adapted with permission from Ref. 1. Copyright 2023 American Chemical Society.

Table S2. Confidence Interval Table for lower and upper sigma levels from 1- to 3-$\boldsymbol{\sigma}$ for the relaxed model.

	3-Sigma-Level	2-Sigma-Level	1-Sigma-Level	Best	1-Sigma-Level	2-Sigma-Level	3-Sigma-Level
	99.730	95.450	68.269	0.000	68.269	95.450	99.730
**gaussian_amplitude_1**	0.047	0.054	0.063	0.074	0.087	0.101	0.117
**gaussian_center_1**	7111.861	7111.890	7111.901	7111.927	7111.955	7111.969	7112.019
**gaussian_fwhmg_1**	1.157	1.222	1.287	1.352	1.416	1.481	1.549
**gaussian_amplitude_2**	0.167	0.188	0.208	0.229	0.250	0.272	0.304
**gaussian_center_2**	7112.997	7113.024	7113.043	7113.109	7113.181	7113.214	7113.295
**gaussian_fwhmg_2**	1.968	2.103	2.223	2.335	2.440	2.542	2.668
**gaussian_amplitude_3**	0.006	0.007	0.049	0.061	0.075	0.095	0.129
**gaussian_center_3**	7117.213	7117.249	7117.265	7117.300	7117.342	7117.358	7117.411
**gaussian_fwhmg_3**	1.069	1.162	1.254	1.345	1.437	1.532	1.645
**gaussian_amplitude_4**	0.084	0.135	0.195	0.260	0.690	0.690	0.846
**gaussian_center_4**	7118.322	7118.508	7118.596	7118.714	7118.804	7118.845	7118.932
**gaussian_fwhmg_4**	1.873	2.145	2.383	2.604	2.820	3.038	3.471
**gaussian_amplitude_5**	0.215	0.568	0.910	1.118	1.349	1.694	inf
**gaussian_center_5**	7119.651	7119.954	7120.098	7120.403	7120.808	7121.018	7121.822
**gaussian_fwhmg_5**	2.601	3.860	4.801	5.322	5.874	6.505	6.981
**gaussian_amplitude_6**	0.063	0.104	0.161	0.238	0.346	0.506	0.753
**gaussian_center_6**	7123.646	7124.040	7124.177	7124.509	7124.724	7124.868	7125.076
**gaussian_fwhmg_6**	2.318	2.667	3.008	3.367	3.760	4.225	4.816
**cgaussian_amplitude_7**	1.030	1.035	1.040	1.046	1.054	1.063	1.077
**cgaussian_center_7**	7120.859	7121.104	7121.219	7121.477	7121.627	7122.096	inf
**cgaussian_fwhmg_7**	-inf	9.168	9.558	9.820	10.058	10.311	10.609

Corresponding tables for Figures

Table S3. The corresponding uncertainties of each component of complex **1** for Figure 7.

	init_value	model_value	best_value	error_relative	error_absolute
gaussian_amplitude_1	1.00	0.108187	0.108187	0.025966	24.001092
gaussian_center_1	7112.10	7112.101986	7112.101986	0.035578	0.000500
gaussian_fwhmg_1	1.50	1.642929	1.642929	0.124933	7.604310
gaussian_amplitude_2	1.00	0.325804	0.325804	0.061962	19.018084
gaussian_center_2	7113.84	7113.518164	7113.518164	0.203911	0.002867
gaussian_fwhmg_2	1.50	2.969291	2.969291	0.203278	6.846014
gaussian_amplitude_3	1.00	1.321790	1.321790	1.510934	114.309686
gaussian_center_3	7117.60	7118.254481	7118.254481	0.371525	0.005219
gaussian_fwhmg_3	1.50	3.574190	3.574190	1.129132	31.591258
gaussian_amplitude_4	1.00	0.075124	0.075124	0.154589	205.779578
gaussian_center_4	7119.60	7119.517696	7119.517696	0.196183	0.002756
gaussian_fwhmg_4	1.50	1.633468	1.633468	0.640066	39.184490
gaussian_amplitude_5	1.00	0.548305	0.548305	1.841444	335.842932
gaussian_center_5	7121.60	7120.803760	7120.803760	0.815264	0.011449
gaussian_fwhmg_5	1.50	2.933822	2.933822	2.294531	78.209615
gaussian_amplitude_6	1.00	2.311901	2.311901	49.485465	2140.466359
gaussian_center_6	7123.60	7122.970122	7122.970122	2.042779	0.028679
gaussian_fwhmg_6	1.50	5.499976	5.499976	16.125059	293.184164
cgaussian_amplitude_7	0.83	1.045161	1.045161	0.108706	10.400870
cgaussian_center_7	7125.00	7124.054569	7124.054569	46.570515	0.653708
cgaussian_fwhmg_7	1.00	6.973078	6.973078	49.437119	708.971266

Table S4. List of uncertainties for runs 0-12, Figure 10 of Complex 1, if calculable.

component	parameter	0	1	2	3	4	5	6	7	8	9	10	11	12
gaussian_amplitude_1	init_value	1	1	1	1	1	1	1	1	1	1	1	1	1
	model_value	0.074276	0.163435	0.041189	7.71E-11	0.179968	0.180071	0.082557	0.082551	0.184569	0.163245	0.175542	0.182088	0.184569
	best_value	0.074276	0.163435	0.041189	7.71E-11	0.179968	0.180071	0.082557	0.082551	0.184569	0.163245	0.175542	0.182088	0.184569
	error_relative	0.014069		0.013577		0.040246	0.040191	0.032811	0.032152	0.035778		0.048739	0.037094	0.035778
	error_absolute	18.94114		32.96283		22.36289	22.31932	39.74388	38.9477	19.38454		27.76491	20.37147	19.38449
gaussian_center_1	init_value	7112	7112	7112	7112	7112	7112	7112	7112	7112	7112	7112	7112	7112
	model_value	7111.994	7112.098	7112.001	7112	7112.111	7112.111	7112.007	7112.007	7112.123	7112.098	7112.099	7112.117	7112.123
	best_value	7111.994	7112.098	7112.001	7112	7112.111	7112.111	7112.007	7112.007	7112.123	7112.098	7112.099	7112.117	7112.123

	error_relative	0.024813		0.031976		0.123301	0.123221	0.061642	0.059	0.111426		0.140104	0.114758	0.111426
	error_absolute	0.000349		0.00045		0.001734	0.001733	0.000867	0.00083	0.001567		0.00197	0.001614	0.001567
gaussian_fwhmg_1	init_value	1.5	1.5	1.5	1.5	1.5	1.5	1.5	1.5	1.5	1.1	1.5	1.5	1.5
	model_value	1.425358	1.723258	1.207838	5.488766	1.683148	1.683407	1.473039	1.472997	1.691898	1.722691	1.678319	1.686684	1.691898
	best_value	1.425358	1.723258	1.207838	5.488766	1.683148	1.683407	1.473039	1.472997	1.691898	1.722691	1.678319	1.686684	1.691898
	error_relative	0.100993		0.188595		0.100697	0.10068	0.197842	0.194723	0.091478		0.110972	0.093844	0.091478
	error_absolute	7.085423		15.61422		5.982647	5.980741	13.43088	13.21955	5.406812		6.612114	5.563801	5.40681
gaussian_amplitude_2	init_value	1	1	1	1	1	1	1	1	1	1	1	1	1
	model_value	0.319012	0.219196	0.325821	0.372843	0.182565	0.182437	0.323112	0.323117	0.179419	0.219385	0.206623	0.179971	0.179419
	best_value	0.319012	0.219196	0.325821	0.372843	0.182565	0.182437	0.323112	0.323117	0.179419	0.219385	0.206623	0.179971	0.179419
	error_relative	0.015817		0.015192		0.050519	0.05048	0.077932	0.208418	0.047998		0.063801	0.047968	0.047998
	error_absolute	4.95813		4.662748		27.6717	27.66964	24.11931	64.50213	26.75184		30.87796	26.65311	26.75178
gaussian_center_2	init_value	7113.6	7113.6	7113.6	7113.6	7113.6	7113.6	7113.6	7113.6	7113.6	7113.6	7113.6	7113.6	7113.6
	model_value	7113.233	7113.592	7112.995	7112.825	7113.564	7113.564	7113.31	7113.31	7113.575	7113.591	7113.593	7113.567	7113.575
	best_value	7113.233	7113.592	7112.995	7112.825	7113.564	7113.564	7113.31	7113.31	7113.575	7113.591	7113.593	7113.567	7113.575
	error_relative	0.061457		0.065167		0.122009	0.121728	0.353267	0.463863	0.093629		0.134688	0.105125	0.093628
	error_absolute	0.000864		0.000916		0.001715	0.001711	0.004966	0.006521	0.001316		0.001893	0.001478	0.001316
gaussian_fwhmg_2	init_value	1.5	1.5	1.5	1.5	1.5	1.5	1.5	1.5	1.5	1.2	1.5	1.5	1.5
	model_value	2.77514	2.214629	2.694175	2.75654	1.814529	1.813729	2.796771	2.796781	1.772189	2.21546	1.909092	1.790499	1.772189
	best_value	2.77514	2.214629	2.694175	2.75654	1.814529	1.813729	2.796771	2.796781	1.772189	2.21546	1.909092	1.790499	1.772189
	error_relative	0.076921		0.075647		0.328953	0.328648	0.284233	0.332064	0.309473		0.411855	0.312506	0.309473
	error_absolute	2.771805		2.80779		18.12881	18.12	10.16291	11.87306	17.46277		21.57332	17.45359	17.46274
gaussian_amplitude_3	init_value	1	1	1	1	1	1	1	1	1	1	1	1	1
	model_value	0.027013	0.093448	3.342325	2.963079	0.027125	0.02715	6.04E-10	3.96E-09	0.035468	0.093444	0.037371	0.030884	0.035468
	best_value	0.027013	0.093448	3.342325	2.963079	0.027125	0.02715	6.04E-10	3.96E-09	0.035468	0.093444	0.037371	0.030884	0.035468

	error_relative	0.005215		0.774261		0.014139	0.014062	0.055325	0.70435	0.017921		0.018082	0.01567	0.017921
	error_absolute	19.3067		23.16534		52.12505	51.79329	9.17E+09	1.78E+10	50.52761		48.38424	50.73907	50.52729
gaussian_center_3	init_value	7117.6	7115.6	7119.6	7119.6	7115.6	7114.6	7115.6	7115.6	7115.6	7115.6	7115.6	7115.6	7115.6
	model_value	7117.363	7117	7119.853	7119.6	7115.046	7115.046	7115.251	7115.478	7115.046	7117	7115.097	7115.047	7115.046
	best_value	7117.363	7117	7119.853	7119.6	7115.046	7115.046	7115.251	7115.478	7115.046	7117	7115.097	7115.047	7115.046
	error_relative	0.036474		0.488558		0.090809	0.090885	45359800	1E+08	0.104994		0.084516	0.097708	0.104995
	error_absolute	0.000512		0.006862		0.001276	0.001277	637501	1409021	0.001476		0.001188	0.001373	0.001476
gaussian_fwhmg_3	init_value	1.5	1.5	1.5	1.5	1.5	1.5	1.5	1.5	1.5	1.5	1.5	1.5	1.5
	model_value	1.129113	2.505753	5.456916	5.261284	1.0913	1.091554	2.727916	3.447861	1.206075	2.505729	1.127301	1.155617	1.206074
	best_value	1.129113	2.505753	5.456916	5.261284	1.0913	1.091554	2.727916	3.447861	1.206075	2.505729	1.127301	1.155617	1.206074
	error_relative	0.121835		0.404641		0.206713	0.205819	28181028	1.16E+08	0.213568		0.17194	0.211169	0.213568
	error_absolute	10.79036		7.415196		18.94193	18.8556	1.03E+09	3.35E+09	17.70772		15.25232	18.27331	17.70769
gaussian_amplitude_4	init_value	1	1	1	1	1	1	1	1	1	1	1	1	1
	model_value	2.293712	3.274217	0.316583	1.912827	0.028865	0.028865	0.90577	0.905771	0.022479	9.33E-09	0.628611	0.027746	0.022479
	best_value	2.293712	3.274217	0.316583	1.912827	0.028865	0.028865	0.90577	0.905771	0.022479	9.33E-09	0.628611	0.027746	0.022479
	error_relative	0.876287		0.568479		0.006898	0.006359	1.118545	9.65068	0.007075		0.157252	0.006446	0.007075
	error_absolute	38.20388		179.5673		23.89915	22.02949	123.4911	1065.466	31.47437		25.01575	23.23139	31.4733
gaussian_center_4	init_value	7119.6	7121.6	7121.6	7121.6	7117.6	7117.6	7117.6	7118.6	7117.6	7117.6	7117.6	7117.6	7117.6
	model_value	7119.237	7120	7122.031	7123.349	7117.337	7117.337	7118.243	7118.243	7117.259	7116.322	7117.6	7117.292	7117.259
	best_value	7119.237	7120	7122.031	7123.349	7117.337	7117.337	7118.243	7118.243	7117.259	7116.322	7117.6	7117.292	7117.259
	error_relative	0.466539		0.537113		0.04398	0.042422	0.362323	0.705207	0.04454		0	0.040563	0.04454
	error_absolute	0.006553		0.007542		0.000618	0.000596	0.000509	0.009907	0.000626		0	0.000057	0.000626
gaussian_fwhmg_4	init_value	1.5	1.5	1.5	1.5	1.5	1.5	1.5	1.5	1.5	1.5	1.5	1.5	1.5
	model_value	4.823323	5.317442	2.589616	4.467462	1.220715	1.220716	3.88305	3.883055	1.092858	5.27023	3.295771	1.178968	1.092857
	best_value	4.823323	5.317442	2.589616	4.467462	1.220715	1.220716	3.88305	3.883055	1.092858	5.27023	3.295771	1.178968	1.092857

	error_relative	0.286514		0.797846		0.148409	0.14299	0.877478	4.630846	0.155117		0.072096	0.137909	0.155115
	error_absolute	5.940186		30.80942		12.15752	11.71361	22.59766	119.2578	14.19374		2.187527	11.69742	14.19351
**gaussian_amplitude_5**	init_value	1	1	1	1	1	1	1	1	1	1	1	1	1
	model_value	3.776543	1.457383	1.2547	9.49E-10	2.288892	2.288887	2.60494	2.60494	1.761802	1.33E-10	1.662536	2.314205	1.761748
	best_value	3.776543	1.457383	1.2547	9.49E-10	2.288892	2.288887	2.60494	2.60494	1.761802	1.33E-10	1.662536	2.314205	1.761748
	error_relative	6.590324		6.881028		0.76823	0.133552	3.805373	60.42928	4.889746		2.786893	0.353359	4.89013
	error_absolute	174.5068		548.4202		33.56341	5.834785	146.0829	2319.796	277.5424		167.629	15.26915	277.5727
**gaussian_center_5**	init_value	7123.6	7123.6	7123.6	7123.6	7119.6	7119.6	7119.6	7119.6	7119.6	7118.2	7119.6	7119.6	7119.6
	model_value	7123.547	7123.493	7123.954	7123.483	7119.233	7119.233	7121	7121	7118.849	7120.52	7119.861	7119.242	7118.849
	best_value	7123.547	7123.493	7123.954	7123.483	7119.233	7119.233	7121	7121	7118.849	7120.52	7119.861	7119.242	7118.849
	error_relative	2.262513		0.723814		0.409653	0.089546	0.973562	13.811	1.907493		0.742019	0.19408	1.907435
	error_absolute	0.031761		0.01016		0.005754	0.001258	0.013672	0.193947	0.026795		0.010422	0.002726	0.026794
**gaussian_fwhmg_5**	init_value	1.5	1.5	1.5	1.5	1.5	1.5	1.5	1.5	1.5	1.2	1.5	1.5	1.5
	model_value	5.499999	3.920047	3.644531	0.972797	4.821806	4.821802	5.5	5.5	4.461045	0.773188	4.107331	4.787275	4.461035
	best_value	5.499999	3.920047	3.644531	0.972797	4.821806	4.821802	5.5	5.5	4.461045	0.773188	4.107331	4.787275	4.461035
	error_relative	2.867451		2.769348		0.256441	0.090151	4830.849	22.27246	1.09806		1.175547	0.13995	1.098177
	error_absolute	52.13549		75.98639		5.318361	1.869662	87833.62	404.9537	24.61442		28.62071	2.923377	24.61709
**cgaussian_amplitude_6**	init_value	0.83	0.83	0.83	0.83									
	model_value	1.028313	1.013218	1.019227	1.019464									
	best_value	1.028313	1.013218	1.019227	1.019464									
	error_relative	0.01494		0.013233										
	error_absolute	1.452892		1.298289										
**cgaussian_center_6**	init_value	7125	7125	7125	7125									
	model_value	7125.693	7124.36	7124.462	7124.431									
	best_value	7125.693	7124.36	7124.462	7124.431									

	error_relative	5.511896		7.404156										
	error_absolute	0.077352		0.103926										
cgaussian_fwhmg_6	init_value	0.8	0.8	0.8	0.8									
	model_value	5	4.508355	4.708169	5									
	best_value	5	4.508355	4.708169	5									
	error_relative	2.245482		7.212346										
	error_absolute	44.90964		153.1879										
gaussian_amplitude_6	init_value					1	1			1	1	1	1	1
	model_value					3.77175	3.771749			3.472172	3.274221	3.893852	0.024372	3.472533
	best_value					3.77175	3.771749			3.472172	3.274221	3.893852	0.024372	3.472533
	error_relative					5.703512	0.411621			26.88246		16.25855	0.006103	26.88297
	error_absolute					151.2166	10.91327			774.226		417.544	25.04189	774.1601
gaussian_center_6	init_value					7123.6	7123.6			7121.6	7121.6	7121.6	7121.6	7121.6
	model_value					7123.538	7123.538			7122.687	7120	7123.619	7121.6	7122.688
	best_value					7123.538	7123.538			7122.687	7120	7123.619	7121.6	7122.688
	error_relative					1.951982	0.293831			9.711699		4.971777	0	9.711674
	error_absolute					0.027402	0.004125			0.136349		0.069793	0	0.136348
gaussian_fwhmg_6	init_value					1.5	1.5			1.5	1.5	1.5	1.5	1.5
	model_value					5.499999	5.5			5.49973	5.317439	5.5	1.5	5.49995
	best_value					5.499999	5.5			5.49973	5.317439	5.5	1.5	5.49995
	error_relative					2.497979	2322.331			16.37566		0.619177	0	12.9469
	error_absolute					45.4178	42224.2			297.754		11.25776	0	235.4004
cgaussian_amplitude_7	init_value					0.83	0.83					0.83		
	model_value					1.028055	1.028055					1.03213		
	best_value					1.028055	1.028055					1.03213		

	error_relative	0.012659	0.006279			0.025161
	error_absolute	1.231327	0.610725			2.437812
cgaussian_center_7	init_value	7125	7125			7125
	model_value	7125.682	7125.682			7125.819
	best_value	7125.682	7125.682			7125.819
	error_relative	4.76405	0.480546			13.1179
	error_absolute	0.066857	0.006744			0.18409
cgaussian_fwhmg_7	init_value	0.8	0.8			0.8
	model_value	5	5			5
	best_value	5	5			5
	error_relative	3.879911	0.028498			26.16635
	error_absolute	77.59823	0.569952			523.327
erf_amplitude_6	init_value			0.83	0.83	
	model_value			1.001203	1.001203	
	best_value			1.001203	1.001203	
	error_relative			0.004281	0.006873	
	error_absolute			0.427601	0.686501	
erf_center_6	init_value			7125	7125	
	model_value			7123	7123	
	best_value			7123	7123	
	error_relative			248.1898	65.33739	
	error_absolute			3.484343	0.917274	
erf_sigma_6	init_value			0.8	0.8	
	model_value			3.154893	3.154892	
	best_value			3.154893	3.154892	
	error_relative			1.329065	10.0316	

	error_absolute	42.12711	317.9696					
gaussian_amplitude_7	init_value			1	1		1	1
	model_value			3.501686	1.457408		3.860867	3.501346
	best_value			3.501686	1.457408		3.860867	3.501346
	error_relative			23.77884			2.624829	23.77851
	error_absolute			679.0683			67.98548	679.125
gaussian_center_7	init_value			7123.6	7123.6		7123.6	7123.6
	model_value			7126.343	7123.493		7123.651	7126.343
	best_value			7126.343	7123.493		7123.651	7126.343
	error_relative			7.385363			0.9207	7.384325
	error_absolute			0.103635			0.012925	0.10362
gaussian_fwhmg_7	init_value			1.5	1.5		1.5	1.5
	model_value			5.021959	3.920041		5.5	5.021821
	best_value			5.021959	3.920041		5.5	5.021821
	error_relative			8.90457			1724.264	8.902922
	error_absolute			177.3127			31350.26	177.2847
erf_amplitude_8	init_value			0.83	0.83			
	model_value			0.966292	1.013218			
	best_value			0.966292	1.013218			
	error_relative			0.290082				
	error_absolute			30.02009				
erf_center_8	init_value			7125	7125			
	model_value			7128.095	7124.36			
	best_value			7128.095	7124.36			
	error_relative			1.201782				

	error_absolute	0.01686				
erf_sigma_8	init_value	0.8	0.8			
	model_value	2.201556	2.707523			
	best_value	2.201556	2.707523			
	error_relative	1.197866				
	error_absolute	54.41				
cgaussian_amplitude_8	init_value				0.83	0.83
	model_value				1.032059	0.966297
	best_value				1.032059	0.966297
	error_relative				0.009489	0.290044
	error_absolute				0.919433	30.01608
cgaussian_center_8	init_value				7125	7125
	model_value				7125.836	7128.095
	best_value				7125.836	7128.095
	error_relative				2.208585	1.201775
	error_absolute				0.030994	0.01686
cgaussian_fwhmg_8	init_value				0.8	0.8
	model_value				5	3.665797
	best_value				5	3.665797
	error_relative				0.404532	1.994314
	error_absolute				8.090637	54.4033

Table S5. List of complex **2** uncertainties for runs 0-1, Figure 11.

component	parameter	0	1
gaussian_amplitude_1	init_value	1	1
	model_value	0.244911	0.245529
	best_value	0.244911	0.245529
	error_relative	0.01427	0.007624
	error_absolute	5.826491	3.105116
gaussian_center_1	init_value	7112	7112
	model_value	7112.313	7112.315
	best_value	7112.313	7112.315
	error_relative	0.034527	0.025105
	error_absolute	0.000485	0.000353
gaussian_fwhmg_1	init_value	1.5	1.5
	model_value	1.814092	1.816197
	best_value	1.814092	1.816197
	error_relative	0.04415	0.037661
	error_absolute	2.43371	2.073619
gaussian_amplitude_2	init_value	1	1
	model_value	0.077066	0.076266
	best_value	0.077066	0.076266
	error_relative	0.020547	0.01684
	error_absolute	26.6614	22.08059
gaussian_center_2	init_value	7113.6	7113.6
	model_value	7113.658	7113.656
	best_value	7113.658	7113.656
	error_relative	0.035347	0.034891
	error_absolute	0.000497	0.00049
gaussian_fwhmg_2	init_value	1.5	1.5
	model_value	1.125491	1.119355
	best_value	1.125491	1.119355
	error_relative	0.089235	0.087098
	error_absolute	7.928581	7.781052
gaussian_amplitude_3	init_value	1	1
	model_value	0.130244	0.136025
	best_value	0.130244	0.136025
	error_relative	0.117413	0.072258
	error_absolute	90.14863	53.121
gaussian_center_3	init_value	7115.6	7115.6
	model_value	7115.065	7115.066
	best_value	7115.065	7115.066
	error_relative	0.048313	0.047133
	error_absolute	0.000679	0.000662
gaussian_fwhmg_3	init_value	1.5	1.5
	model_value	1.596062	1.621461

	best_value	1.596062	1.621461
	error_relative	0.349092	0.24648
	error_absolute	21.87206	15.20108
gaussian_amplitude_4	init_value	1	1
	model_value	0.040677	0.03845
	best_value	0.040677	0.03845
	error_relative	0.031313	0.025747
	error_absolute	76.978	66.96111
gaussian_center_4	init_value	7117.6	7117.6
	model_value	7117.15	7117.139
	best_value	7117.15	7117.139
	error_relative	0.063833	0.054743
	error_absolute	0.000897	0.000769
gaussian_fwhmg_4	init_value	1.5	1.5
	model_value	1.138103	1.112077
	best_value	1.138103	1.112077
	error_relative	0.230351	0.206818
	error_absolute	20.23995	18.59744
gaussian_amplitude_5	init_value	1	1
	model_value	0.825946	0.828115
	best_value	0.825946	0.828115
	error_relative	2.363174	3.039846
	error_absolute	286.1174	367.0801
gaussian_center_5	init_value	7119.6	7119.6
	model_value	7118.043	7117.992
	best_value	7118.043	7117.992
	error_relative	0.210409	0.143256
	error_absolute	0.002956	0.002013
gaussian_fwhmg_5	init_value	1.5	1.5
	model_value	3.103612	3.081632
	best_value	3.103612	3.081632
	error_relative	1.621268	1.700483
	error_absolute	52.2381	55.18127
gaussian_amplitude_6	init_value	1	1
	model_value	2.380456	1.277986
	best_value	2.380456	1.277986
	error_relative	20.53996	8.536941
	error_absolute	862.8579	667.9994
gaussian_center_6	init_value	7121.6	7121.6
	model_value	7120.636	7120.011
	best_value	7120.636	7120.011
	error_relative	8.149621	1.96102
	error_absolute	0.114451	0.027542
gaussian_fwhmg_6	init_value	1.5	1.5
	model_value	5.066929	4.493403

	best_value	5.066929	4.493403
	error_relative	9.384957	8.69618
	error_absolute	185.2198	193.5322
gaussian_amplitude_7	init_value	1	1
	model_value	3.854547	2.586177
	best_value	3.854547	2.586177
	error_relative	39.54403	10.49445
	error_absolute	1025.906	405.7901
gaussian_center_7	init_value	7123.6	7123.6
	model_value	7124.015	7122.758
	best_value	7124.015	7122.758
	error_relative	7.275131	3.930099
	error_absolute	0.102121	0.055177
gaussian_fwhmg_7	init_value	1.5	1.5
	model_value	5.499917	5.5
	best_value	5.499917	5.5
	error_relative	18.7574	1897.918
	error_absolute	341.0488	34507.59
cgaussian_amplitude_8	init_value	0.83	0.83
	model_value	1.134019	1.144425
	best_value	1.134019	1.144425
	error_relative	0.040666	0.048106
	error_absolute	3.585968	4.203528
cgaussian_center_8	init_value	7125	7125
	model_value	7126.17	7124.151
	best_value	7126.17	7124.151
	error_relative	20.44671	9.891991
	error_absolute	0.286924	0.138852
cgaussian_fwhmg_8	init_value	0.8	0.8
	model_value	5	7
	best_value	5	7
	error_relative	10.98852	17.62303
	error_absolute	219.7704	251.7576

Table S6. List of uncertainties for simultaneously fitting, Figure 11.

component	parameter	0
gaussian_amplitude_1_1	init_value	1
	model_value	0.199221
	best_value	0.199221
gaussian_center_1_1	init_value	7112
	model_value	7112.182
	best_value	7112.182
gaussian_fwhmg_1_1	init_value	1.5
	model_value	1.710144

	**best_value**	1.710144
**gaussian_amplitude_2_1**	**init_value**	1
	**model_value**	0.175607
	**best_value**	0.175607
**gaussian_center_2_1**	**init_value**	7113.6
	**model_value**	7113.682
	**best_value**	7113.682
**gaussian_fwhmg_2_1**	**init_value**	1.5
	**model_value**	1.697672
	**best_value**	1.697672
**gaussian_amplitude_3_1**	**init_value**	1
	**model_value**	0.054558
	**best_value**	0.054558
**gaussian_center_3_1**	**init_value**	7115.6
	**model_value**	7115.155
	**best_value**	7115.155
**gaussian_fwhmg_3_1**	**init_value**	1.5
	**model_value**	1.153463
	**best_value**	1.153463
**gaussian_amplitude_4_1**	**init_value**	1
	**model_value**	0.880755
	**best_value**	0.880755
**gaussian_center_4_1**	**init_value**	7116.6
	**model_value**	7117.914
	**best_value**	7117.914
**gaussian_fwhmg_4_1**	**init_value**	1.5
	**model_value**	3.094906
	**best_value**	3.094906
**gaussian_amplitude_5_1**	**init_value**	1
	**model_value**	0.680659
	**best_value**	0.680659
**gaussian_center_5_1**	**init_value**	7118.6
	**model_value**	7120.006
	**best_value**	7120.006
**gaussian_fwhmg_5_1**	**init_value**	1.5
	**model_value**	2.816948
	**best_value**	2.816948
**gaussian_amplitude_6_1**	**init_value**	1
	**model_value**	0.568143
	**best_value**	0.568143
**gaussian_center_6_1**	**init_value**	7120.6
	**model_value**	7122.333
	**best_value**	7122.333
**gaussian_fwhmg_6_1**	**init_value**	1.5
	**model_value**	3.5

	best_value	3.5
gaussian_amplitude_7_1	init_value	1
	model_value	4.999985
	best_value	4.999985
gaussian_center_7_1	init_value	7121.6
	model_value	7124.191
	best_value	7124.191
gaussian_fwhmg_7_1	init_value	1.5
	model_value	0.112529
	best_value	0.112529
gaussian_amplitude_8_1	init_value	1
	model_value	4.998523
	best_value	4.998523
gaussian_center_8_1	init_value	7121.6
	model_value	7124.316
	best_value	7124.316
gaussian_fwhmg_8_1	init_value	1.5
	model_value	0.112005
	best_value	0.112005
cgaussian_amplitude_9_1	init_value	0.83
	model_value	1.016534
	best_value	1.016534
cgaussian_center_9_1	init_value	7121
	model_value	7121.793
	best_value	7121.793
cgaussian_fwhmg_9_1	init_value	1
	model_value	7
	best_value	7
gaussian_amplitude_1_2	init_value	1
	model_value	0.207175
	best_value	0.207175
gaussian_center_1_2	init_value	7112
	model_value	7112.182
	best_value	7112.182
gaussian_fwhmg_1_2	init_value	1.5
	model_value	1.710144
	best_value	1.710144
gaussian_amplitude_2_2	init_value	1
	model_value	0.146925
	best_value	0.146925
gaussian_center_2_2	init_value	7113.6
	model_value	7113.682
	best_value	7113.682
gaussian_fwhmg_2_2	init_value	1.5
	model_value	1.697672

	**best_value**	1.697672
**gaussian_amplitude_3_2**	**init_value**	1
	**model_value**	0.068963
	**best_value**	0.068963
**gaussian_center_3_2**	**init_value**	7115.6
	**model_value**	7115.155
	**best_value**	7115.155
**gaussian_fwhmg_3_2**	**init_value**	1.5
	**model_value**	1.153463
	**best_value**	1.153463
**gaussian_amplitude_4_2**	**init_value**	1
	**model_value**	1.081871
	**best_value**	1.081871
**gaussian_center_4_2**	**init_value**	7116.6
	**model_value**	7117.914
	**best_value**	7117.914
**gaussian_fwhmg_4_2**	**init_value**	1.5
	**model_value**	3.094906
	**best_value**	3.094906
**gaussian_amplitude_5_2**	**init_value**	1
	**model_value**	0.621117
	**best_value**	0.621117
**gaussian_center_5_2**	**init_value**	7118.6
	**model_value**	7120.006
	**best_value**	7120.006
**gaussian_fwhmg_5_2**	**init_value**	1.5
	**model_value**	2.816948
	**best_value**	2.816948
**gaussian_amplitude_6_2**	**init_value**	1
	**model_value**	0.808865
	**best_value**	0.808865
**gaussian_center_6_2**	**init_value**	7120.6
	**model_value**	7122.333
	**best_value**	7122.333
**gaussian_fwhmg_6_2**	**init_value**	1.5
	**model_value**	3.5
	**best_value**	3.5
**gaussian_amplitude_7_2**	**init_value**	1
	**model_value**	1.468935
	**best_value**	1.468935
**gaussian_center_7_2**	**init_value**	7121.6
	**model_value**	7124.191
	**best_value**	7124.191
**gaussian_fwhmg_7_2**	**init_value**	1.5
	**model_value**	0.112529

	**best_value**	0.112529
**gaussian_amplitude_8_2**	**init_value**	1
	**model_value**	2.147344
	**best_value**	2.147344
**gaussian_center_8_2**	**init_value**	7121.6
	**model_value**	7124.316
	**best_value**	7124.316
**gaussian_fwhmg_8_2**	**init_value**	1.5
	**model_value**	0.112005
	**best_value**	0.112005
**cgaussian_amplitude_9_2**	**init_value**	0.83
	**model_value**	1.091819
	**best_value**	1.091819
**cgaussian_center_9_2**	**init_value**	7121
	**model_value**	7121.793
	**best_value**	7121.793
**cgaussian_fwhmg_9_2**	**init_value**	1
	**model_value**	7
	**best_value**	7

Changing the Type of Metric

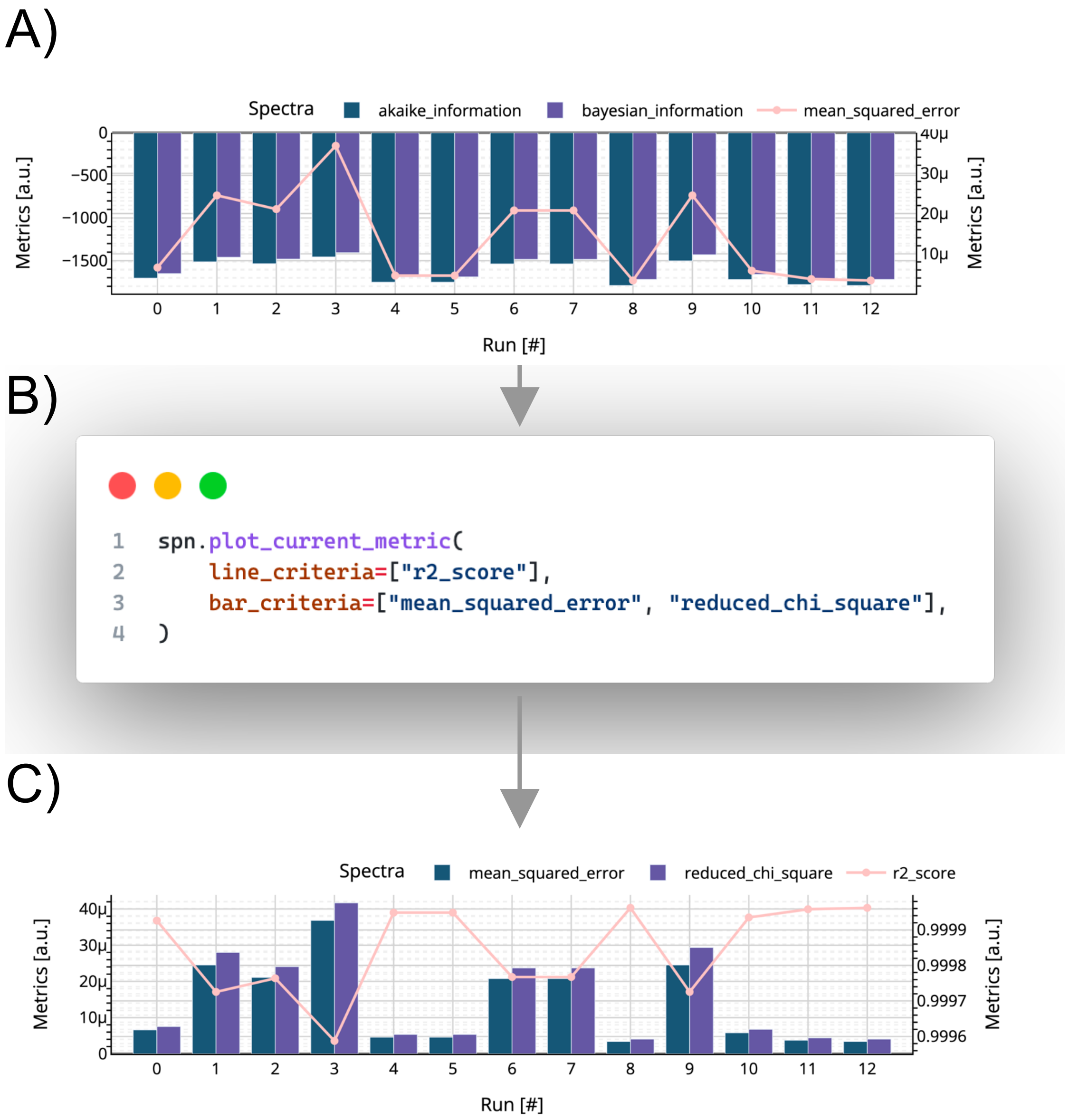

Figure S5. On the top, Plot A displays the metrics as a series of runs with bar plots for Akaike Information and Bayesian Information Criteria, and a line plot for Mean Square Error. In the middle, Plot B shows the corresponding command to switch with one execution according to the new metric for bar and line. On the bottom, Plot C displays the results as a bar plot for Mean Square Error and reduced Chi-Square and a line plot for R^2.

About `DescriptionAPI` and `*.lock files`

As per the Good Scientific Practice Guideline 11 (Method and Standards) and 12 (Documentation) of the Deutsche Forschungs Gemeinschaft (DFG),[7] we have implemented `DescriptionAPI` via Pydantic,[8] which allows native export to Python dictionaries, and from there, to the JSON or toml file format, which can be saved as a `*.lock` file.

The `DescriptionAPI` is automatically activated and it captures significant information such as the hash value of the host, version of SpectraFit, and major libraries.

Additionally, it enables users to store different types of Metadata, including refs, `projectDescription`, or free field definitions like `meta_data`; you can see Figure S6 for more information. Metadata can include details such as the sample, detection, endstation, calibration, or even a "Notice to the Reader" to guide the use of the data for scientific purposes. The notice can also be found at the end of the manuscript and serves to guide the data's use for scientific purposes. In general, metadata plays a crucial role in providing context for future analysis and keeping track of the data's suitability.

In summary, the SpectraFitNotebook object helps users organize their digital work, which is defined by the data itself and the description of the data, meaning meta-data. This allows users to provide all necessary data by simply inspecting the `*.lock` file. Our primary objective is to treat the fitting process as a scientific sub-project with the intention to publish. To achieve this, we have automated the tracking of crucial information, which prevents any chance of data re-engineering or reconstruction.

```
description=DescriptionAPI(
    refs=["https://doi.org/10.1021/acs.inorgchem.2c03693"],
    project_name="SpectraFit Example XAS for 2Fe2C",
    projectDescription="Example of SpectraFit XAS fitting for 2Fe2C",
    projectTags=["XAS", "2Fe2C", "HERFD"],
    authors=["Anselm W. Hahn", "Joseph Zsmobor-Pindera"],
    meta_data={
        "sample_name": "2Fe2C",
        "sample_id": "2Fe2C-1",
        "detection": ["Fe Kβ1,3 high-energy-resolution fluorescence detected XAS "],
        "endstation": [
            "beamline 6-2, Stanford synchrotron radiation lightsource (SSRL)",
            "beamline 9-3, Stanford synchrotron radiation lightsource (SSRL)",
        ],
        "calibration": [
            {"XAS": "First inflection point of Fe set to 7111.2 eV"},
            {"XES": "Maximum of the Kβ1,3 line of an Fe2O3 reference to 7060.6 eV"},
        ],
        "NOTICE_TO_THE_READER": (
            "The referenced material is excerpted from the ACS - Inorganic Chemistry article"
            " 'Sulfur-Ligated [2Fe-2C] Clusters as Synthetic Model Systems for Nitrogenase'"
            " by S. Yogendra et al. The original article can be found at"
            " <https://pubs.acs.org/doi/full/10.1021/acs.inorgchem.2c03693>. Permission"
            " to reuse referenced or this material must be granted by the"
            " American Chemical Society (ACS). Further permissions related"
            " to the material excerpted should be directed to the ACS."
        ),
```

Figure S6. Code capture of the input data for the `DescriptionAPI`.

Post-Processing via Scikit-Learn

When analyzing spectral data, it can be beneficial to represent the actual spectra accurately through True vs. Predicted presentation, as shown in Figure S7. This helps in easily identifying the general accuracy, outliers, and distribution of the values. To support these observations, the bin plots (Figure S7) on top and right sight of the plot further illustrate the distribution. The corresponding source code for how to derive this plot Figure S7 from the corresponding fit results is provided in Figure S4.

In this context, (S_i) represents the actual spectrum values, while $(\widehat{S_i})$ denotes the predicted spectrum values. The scatter plot represents the relationship between the actual and predicted (fitted) spectra with each point $(S_i, \widehat{S_i})$ depicting this relationship.

$$\text{True vs. Predict Plot: } \widehat{S_i} = f(S_i) \quad \forall\, i = 1,2,\ldots,N$$

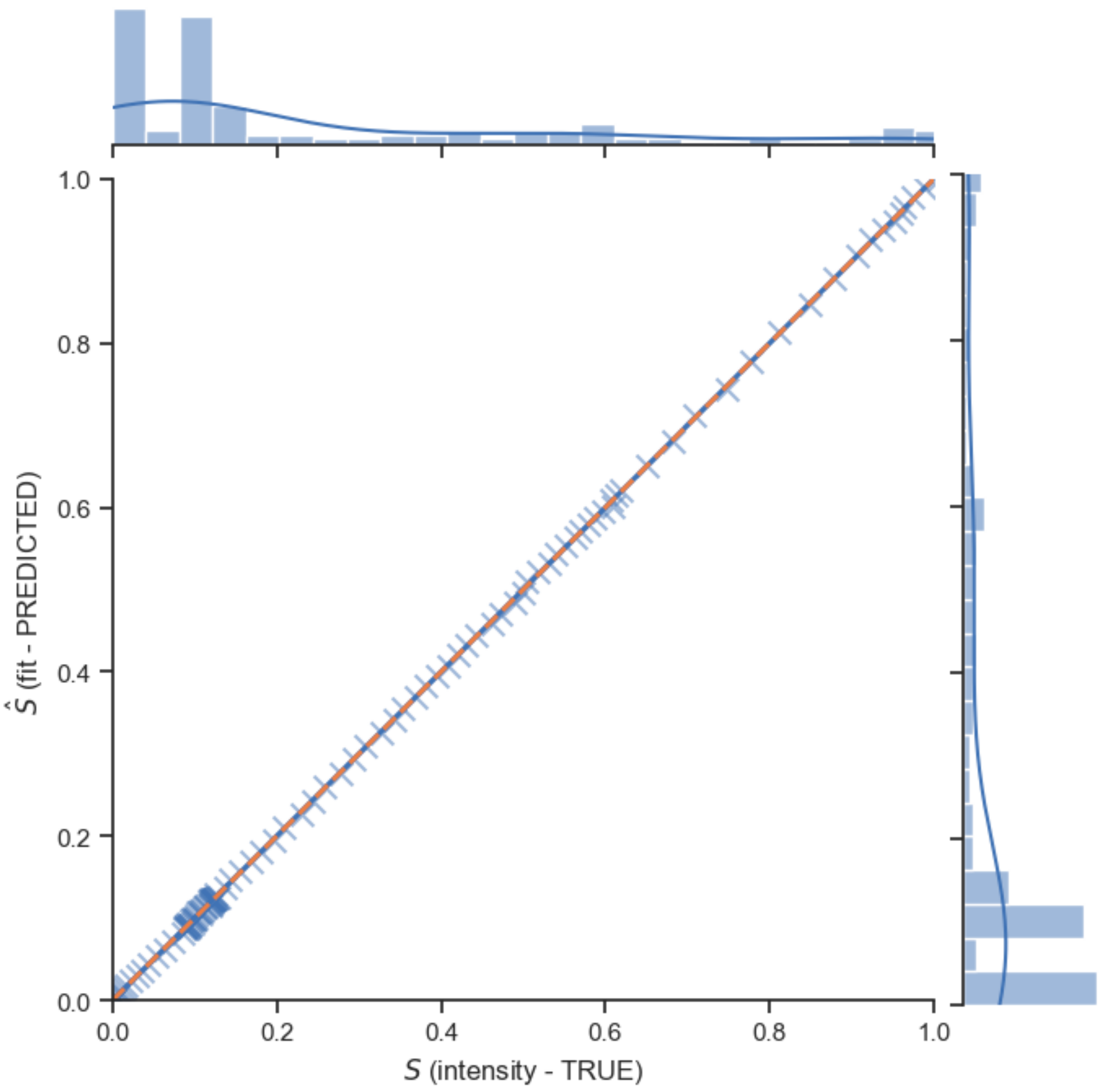

Figure S7. Displaying of a scatter plot that compares the True and Predicted spectral data. The x-axis represents the True values, while the y-axis represents the Predicted values. The scatter plot is shown with blue crosses to indicate the accuracy, outliers, and distribution of values. An orange reference line with a slope of 1 is also provided, which represents ideal conditions where the predicted values are equal to the true values. Moreover, each axis has a corresponding bin plot that shows the density of data points along with the calculated kernel density estimate (KDE).

The comparison between S_i and $\widehat{S_i}$ is the basis that SpectraFit uses to achieve quantitative evaluation through various scikit-learn[9] scoring metrics implemented within the SpectraFit:

1. **Explained Variance Score (*EVS*):**

$$\text{EVS} = 1 - \frac{\text{Var}(S - \hat{S})}{\text{Var}(S)}$$

2. **R-squared (R^2)**

$$R^2 = 1 - \frac{\sum_{i=1}^{N}(S_i - \widehat{S_i})^2}{\sum_{i=1}^{N}(S_i - \bar{S})^2}$$

3. **Max Error:**

$$\text{Max Error} = \max_i |S_i - \widehat{S_i}|$$

4. **Mean Absolute Error (MAE):**

$$\text{MAE} = \frac{1}{N}\sum_{i=1}^{N}|S_i - \widehat{S_i}|$$

5. **Mean Squared Error (MSE):**

$$\text{MSE} = \frac{1}{N}\sum_{i=1}^{N}(S_i - \widehat{S_i})^2$$

6. **Mean Squared Log Error (MSLE):**

$$\text{MSLE} = \frac{1}{N}\sum_{i=1}^{N}\left(\log(1 + S_i) - \log(1 + \widehat{S_i})\right)^2$$

7. **Median Absolute Error (MeAE):**

$$\text{MeAE} = \text{median}_i|S_i - \widehat{S_i}|$$

8. **Mean Absolute Percentage Error (MAPE):**

$$\text{MAPE} = \frac{1}{N}\sum_{i=1}^{N}\left|\frac{S_i - \widehat{S_i}}{S_i}\right| \times 100$$

9. **Mean Poisson Deviance (MPD):**

$$\text{MPD} = \frac{2}{N}\sum_{i=1}^{N}\left(S_i \log\left(\frac{S_i}{\widehat{S_i}}\right) - (S_i - \widehat{S_i})\right)$$

where (S) represents the actual spectrum values, ($\hat{S}$) represents the predicted spectrum values, and ($\bar{S}$) represents the mean of the actual spectrum values.

```python
from pathlib import Path

import matplotlib.pyplot as plt
import numpy as np
import pandas as pd
import seaborn as sns


def plot_true_vs_predicted(
    df: pd.DataFrame,
    x_true: str = "intensity",
    y_predict: str = "fit",
    fname: str = "true_vs_predicted",
) -> None:
    """Plots the true values against the predicted values using a jointplot.

    Args:
        df (pd.DataFrame): The DataFrame containing the data of the final fit.
        x_true (str, optional): The column name for the true values
            on the x-axis. Default is "intensity".
        y_predict (str, optional): The column name for the predicted values
            on the y-axis. Default is "fit".
        fname (str, optional): The filename to save the plot.
            Default is "true_vs_predicted".

    """
    # Set theme
    sns.set_theme(style="ticks")

    # Create a jointplot
    g = sns.jointplot(
        x=x_true,
        y=y_predict,
        data=df,
        kind="reg",
        truncate=True,
        xlim=(0, 1),
        ylim=(0, 1),
        color=sns.color_palette()[0],
        marker="x",
        height=7,
        marginal_kws=dict(bins=25, fill=True, color=sns.color_palette()[0]),
        scatter_kws=dict(s=100, alpha=0.5, color=sns.color_palette()[0]),
    )

    # Add a reference line
    x0, x1 = g.ax_joint.get_xlim()
    y0, y1 = g.ax_joint.get_ylim()
    lims = [max(x0, y0), min(x1, y1)]
    g.ax_joint.plot(lims, lims, "--", color=sns.color_palette()[1], linewidth=2.0)
    g.ax_joint.set_aspect("equal")
    g.ax_joint.set(
        xlabel=f"$S$ ({x_true} - TRUE)",
        ylabel=r"$\hat{S}$" + f" ({y_predict} - PREDICTED)",
    )
    # Save the plot
    plt.savefig(Path(f"{fname}.pdf"), dpi=300)


# Load the data
# df_fit = pd.read_csv("reference_file.csv")
# or use the existing DataFrame in the notebook

# Plot the true vs predicted values
plot_true_vs_predicted(
    df=df_fit, x_true="intensity", y_predict="fit", fname="true_vs_predicted"
)
```

Figure S8. Reference code to generate a True vs. Predicted plot via Seaborn library and save the result as PDF.

Plugins

Based on the open-source philosophy of SpectraFit, the user can extend the functionality of SpectraFit by suggesting new plugins as so-called pull requests. The plugins are implemented as Python modules and are stored in the plugins folder. SpectraFit automatically loads the plugins and can be used by the user. The following plugins are currently available:

- Exporting input and output files
- Working with `*.pkl` files for RIXS data
- Analysis of RIXS data via Graphs
- Visualization of RIXS data via Plotly Dash[10]

CLI vs. Jupyter

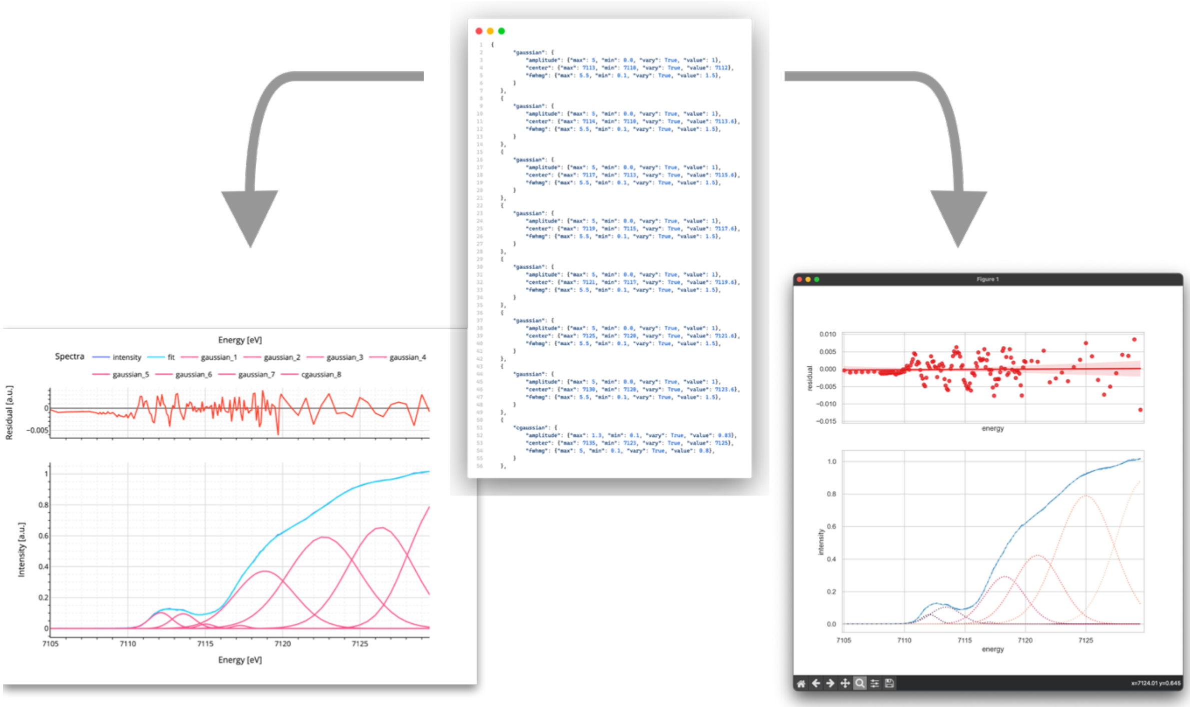

Figure S9. When using SpectraFit, the results are identical regardless of using CLI or Jupyter-mode. Adapted with permission from Ref. 1. Copyright 2023 American Chemical Society.

Fit series of Figure 10 for complex 1

The complete fit series for Figure 10 of complex **1** is shown from Figures S10 to S22. The Figures can be easily replicated by using an additional Jupyter Notebook provided. To use it, SpectraFit needs to be installed in `Jupyter` or `All` mode. This can be done by running either <<**conda install spectrafit-all**>> or <<**pip install spectrafit[all]**>>. It is recommended that a special dedicated environment be used for either `conda` or `CPython`. In case further guidance is needed, the following URL:

- https://anselmoo.github.io/spectrafit/latest/interface/installation/#via-environment

The notebook can be utilized through the built-in Jupyter support of either VSCode or Jetbrains Pycharm or by running the command `jupyter-notebook`. Lastly, SpectraFit can be easily run via Docker by typing `docker run -it -p 8888:8888 ghcr.io/anselmoo/spectrafit:latest`.

It is important to note that the fit results of Figures S18 and S22 appear to be identical, which is consistent with the fact that the main difference between the two is the incorporation of the effect of an edge jump. This change only involves switching the function from an Error-function to a cumulative Gaussian, but the same set of initial parameters and Gaussian functions are used.

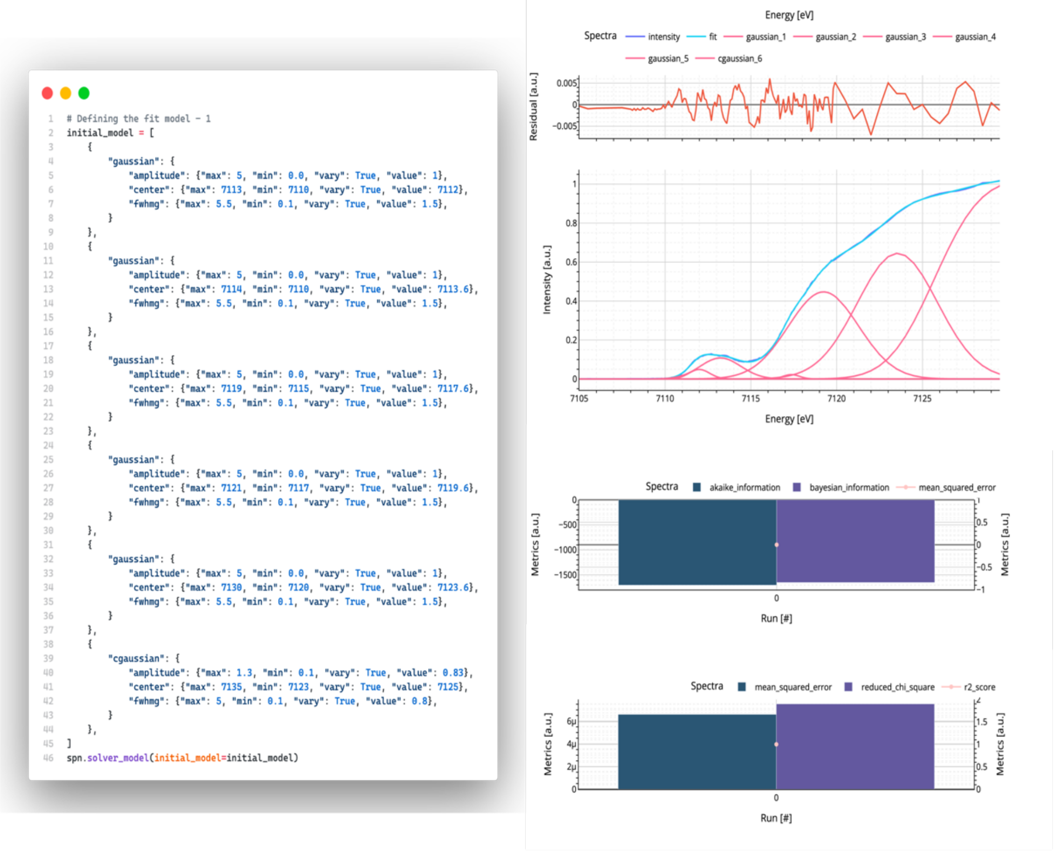

Figure S10. Run 0. Adapted with permission from Ref. 1. Copyright 2023 American Chemical Society.

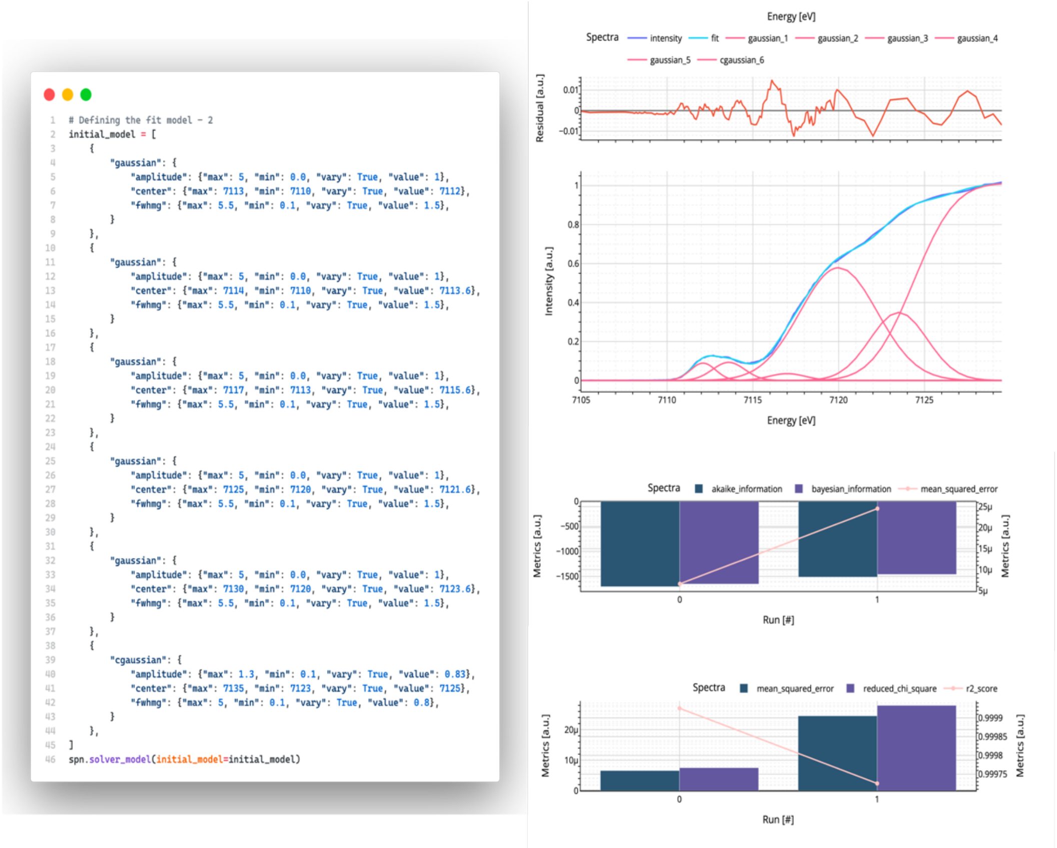

Figure S11. Run 1. Adapted with permission from Ref. 1. Copyright 2023 American Chemical Society.

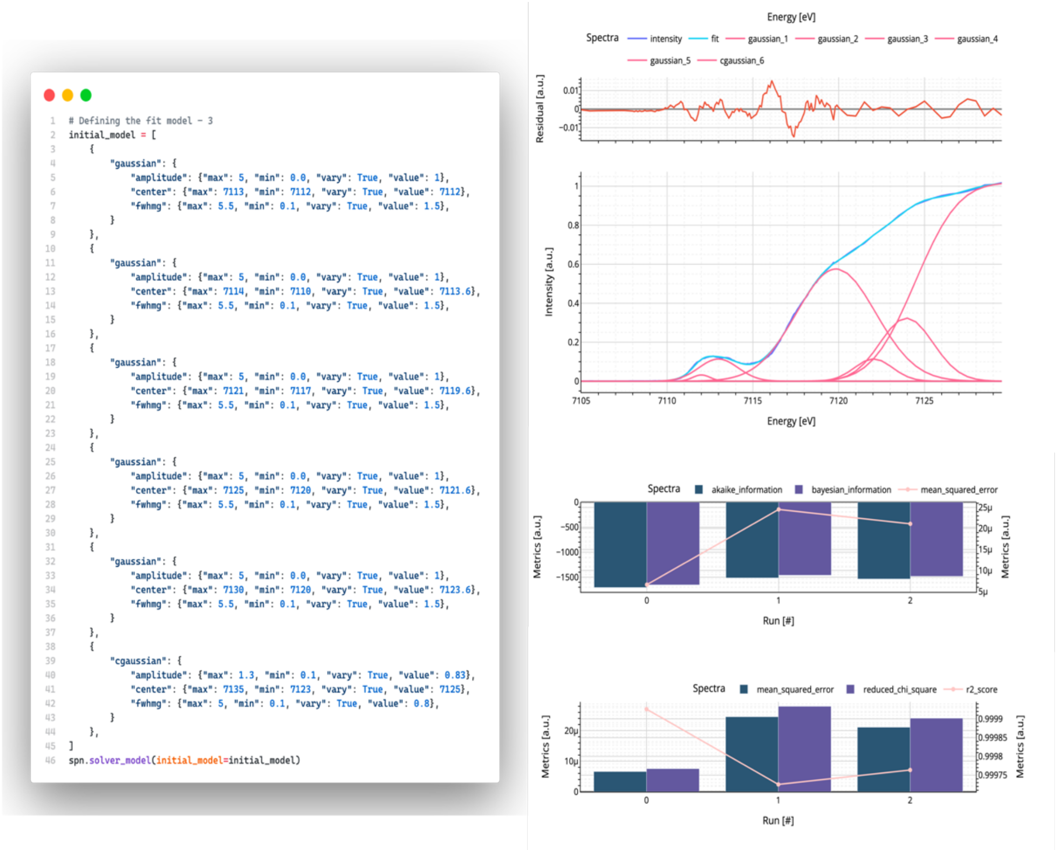

Figure S12. Run 2. Adapted with permission from Ref. 1. Copyright 2023 American Chemical Society.

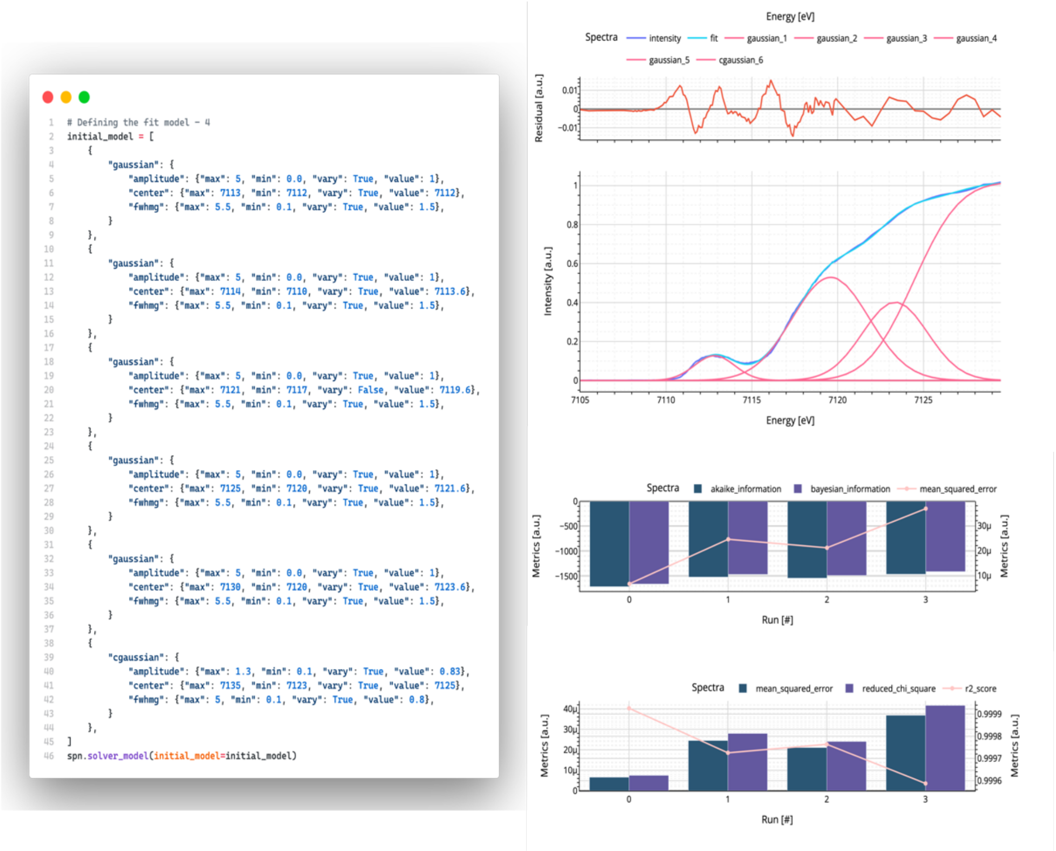

Figure S13. Run 3. Adapted with permission from Ref. 1. Copyright 2023 American Chemical Society.

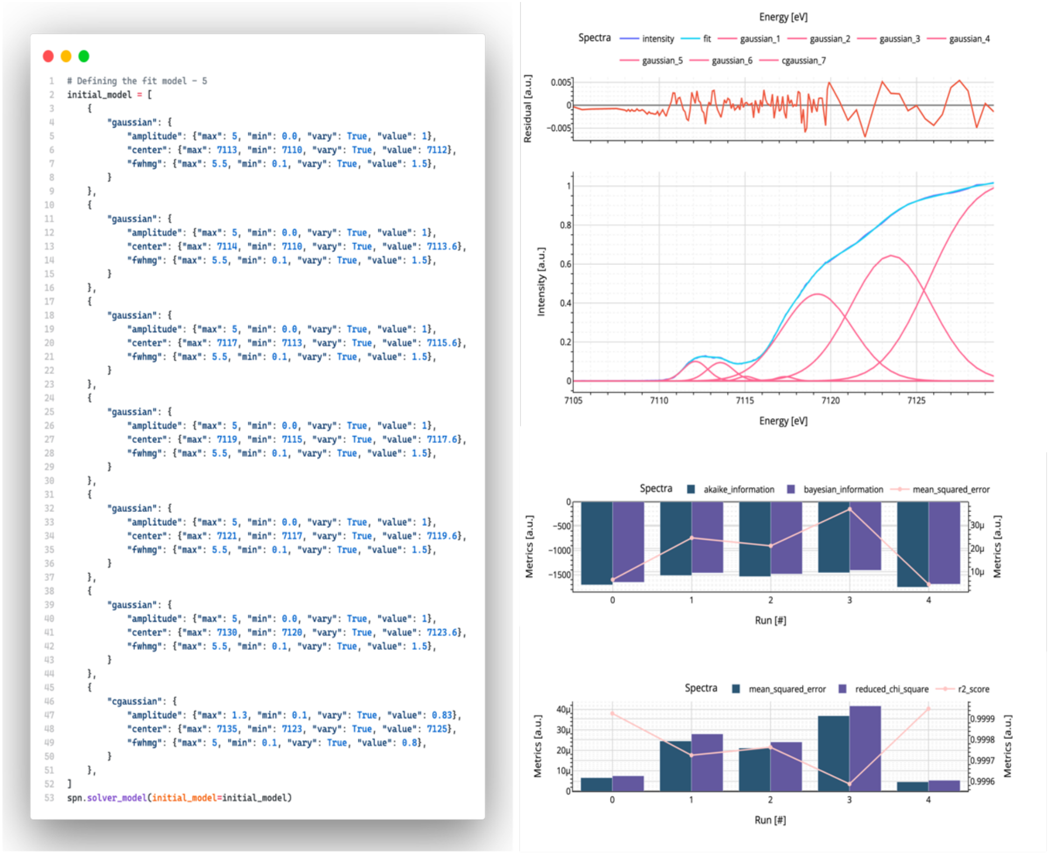

Figure S14. Run 4. Adapted with permission from Ref. 1. Copyright 2023 American Chemical Society.

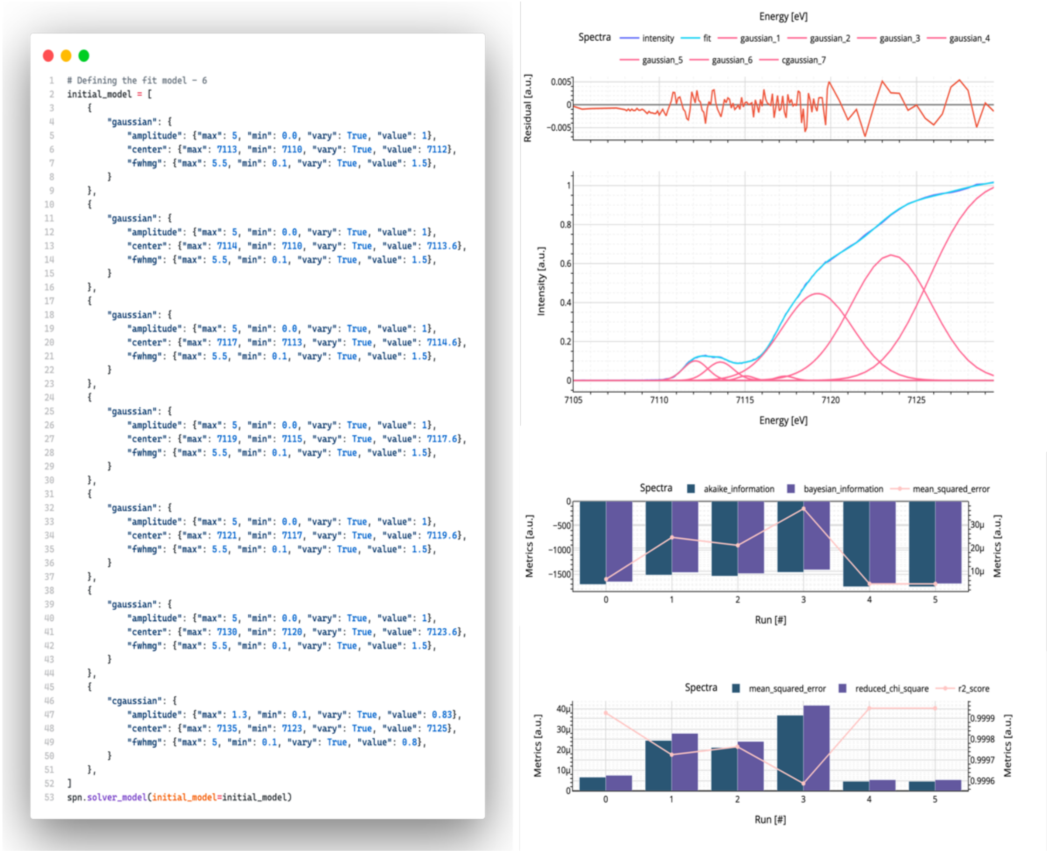

Figure S15. Run 5. Adapted with permission from Ref. 1. Copyright 2023 American Chemical Society.

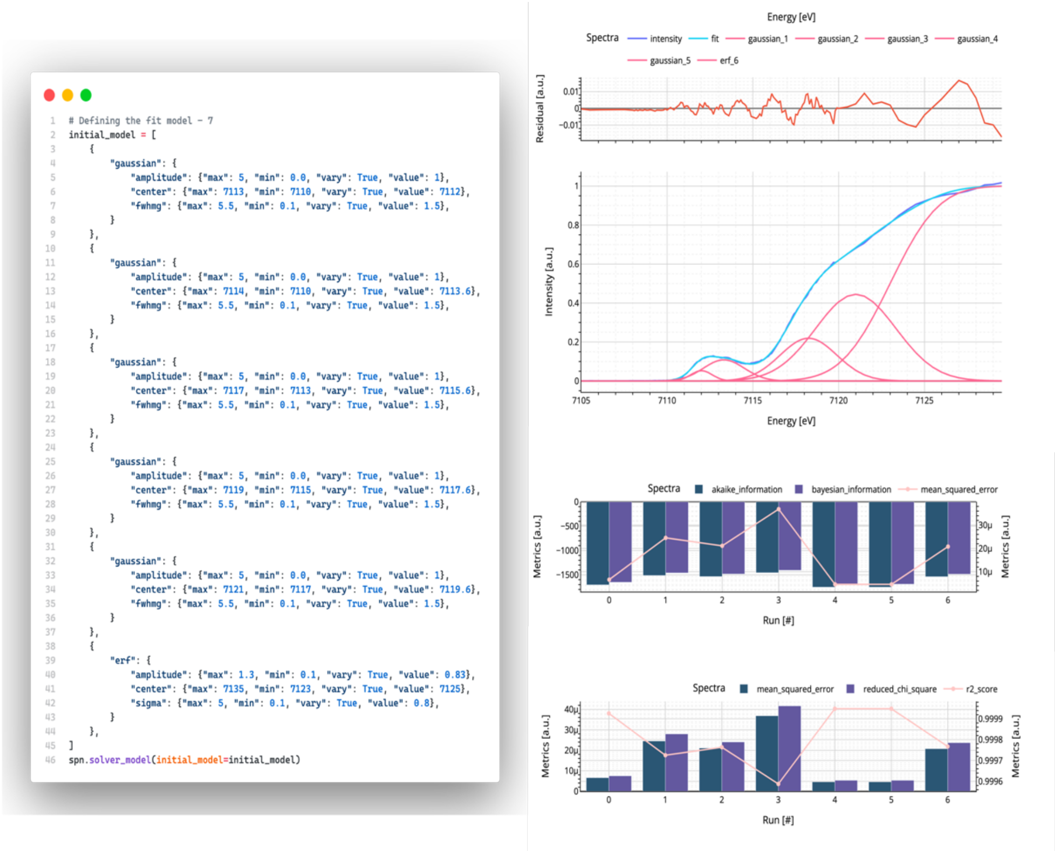

Figure S16. Run 6. Adapted with permission from Ref. 1. Copyright 2023 American Chemical Society.

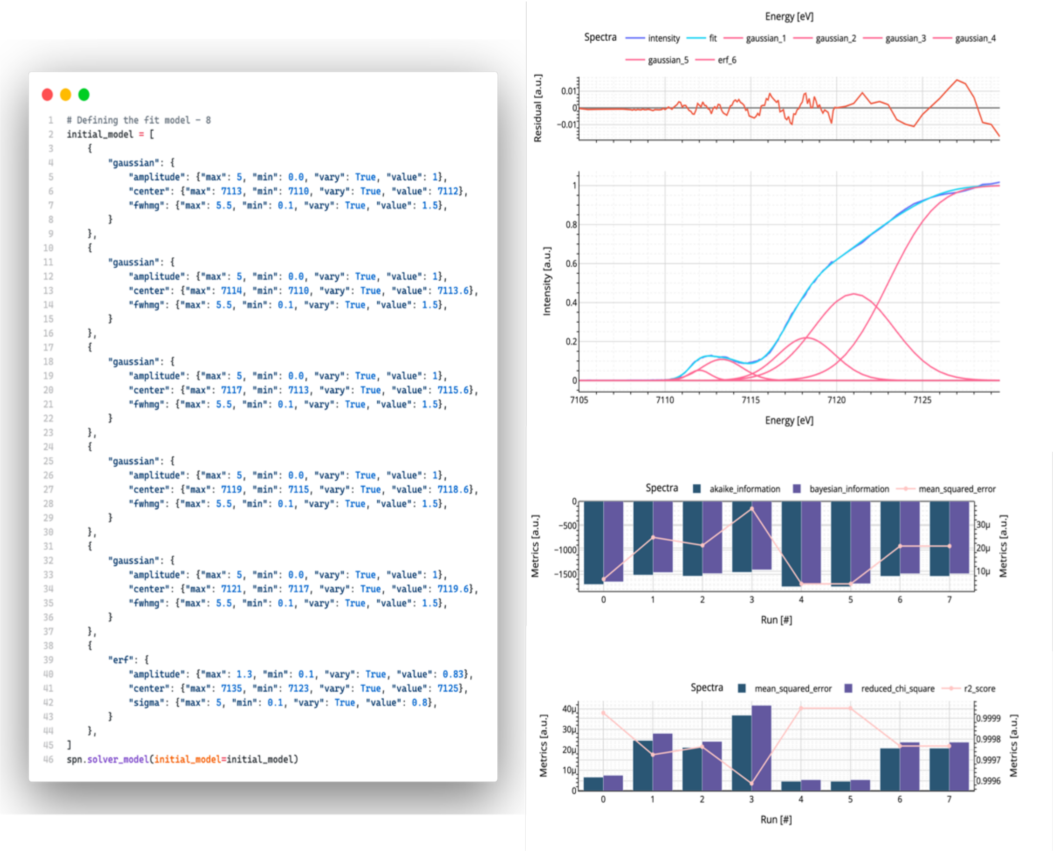

Figure S17. Run 7. Adapted with permission from Ref. 1. Copyright 2023 American Chemical Society.

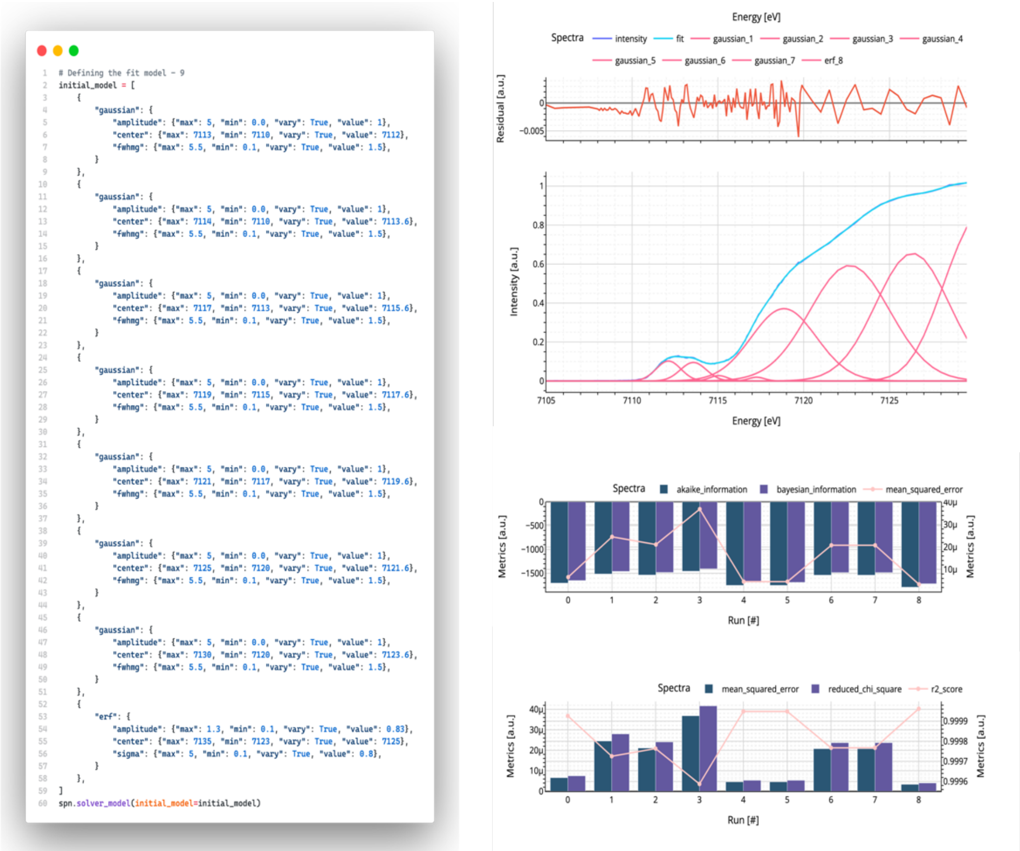

Figure S18. Run 8. Adapted with permission from Ref. 1. Copyright 2023 American Chemical Society.

```
# Defining the fit model - 10
initial_model = [
    {
        "gaussian": {
            "amplitude": {"max": 5, "min": 0.0, "vary": True, "value": 1},
            "center": {"max": 7113, "min": 7110, "vary": True, "value": 7112},
            "fwhmg": {"max": 5.5, "min": 0.1, "vary": True, "value": 1.1},
        }
    },
    {
        "gaussian": {
            "amplitude": {"max": 5, "min": 0.0, "vary": True, "value": 1},
            "center": {"max": 7114, "min": 7110, "vary": True, "value": 7113.6},
            "fwhmg": {"max": 5.5, "min": 0.1, "vary": True, "value": 1.2},
        }
    },
    {
        "gaussian": {
            "amplitude": {"max": 5, "min": 0.0, "vary": True, "value": 1},
            "center": {"max": 7117, "min": 7113, "vary": True, "value": 7115.6},
            "fwhmg": {"max": 5.5, "min": 0.1, "vary": True, "value": 1.5},
        }
    },
    {
        "gaussian": {
            "amplitude": {"max": 5, "min": 0.0, "vary": True, "value": 1},
            "center": {"max": 7119, "min": 7115, "vary": True, "value": 7117.6},
            "fwhmg": {"max": 5.5, "min": 0.1, "vary": True, "value": 1.5},
        }
    },
    {
        "gaussian": {
            "amplitude": {"max": 5, "min": 0.0, "vary": True, "value": 1},
            "center": {"max": 7121, "min": 7117, "vary": True, "value": 7118.2},
            "fwhmg": {"max": 5.5, "min": 0.1, "vary": True, "value": 1.2},
        }
    },
    {
        "gaussian": {
            "amplitude": {"max": 5, "min": 0.0, "vary": True, "value": 1},
            "center": {"max": 7125, "min": 7120, "vary": True, "value": 7121.6},
            "fwhmg": {"max": 5.5, "min": 0.1, "vary": True, "value": 1.5},
        }
    },
    {
        "gaussian": {
            "amplitude": {"max": 5, "min": 0.0, "vary": True, "value": 1},
            "center": {"max": 7130, "min": 7120, "vary": True, "value": 7123.6},
            "fwhmg": {"max": 5.5, "min": 0.1, "vary": True, "value": 1.5},
        }
    },
    {
        "erf": {
            "amplitude": {"max": 1.3, "min": 0.1, "vary": True, "value": 0.83},
            "center": {"max": 7135, "min": 7123, "vary": True, "value": 7125},
            "sigma": {"max": 5, "min": 0.1, "vary": True, "value": 0.8},
        }
    },
]
spn.solver_model(initial_model=initial_model)
```

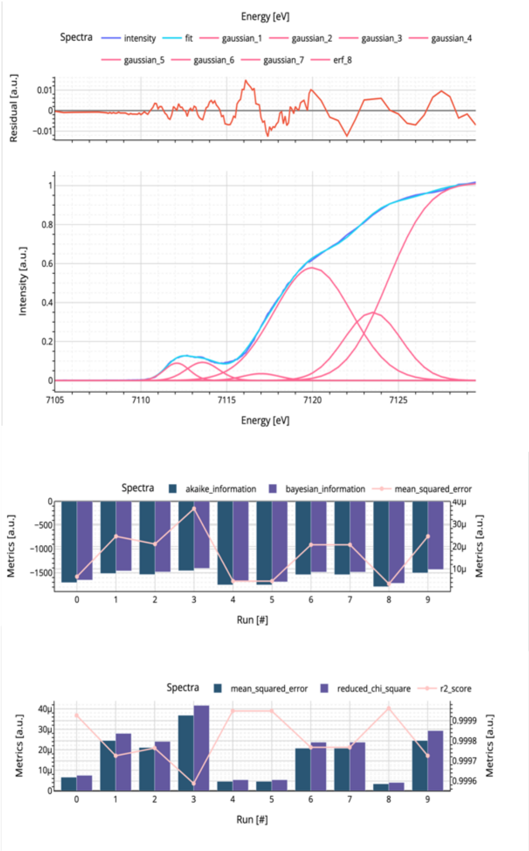

Figure S19. Run 9. Adapted with permission from Ref. 1. Copyright 2023 American Chemical Society.

```
# Defining the fit model - 11
initial_model = [
    {
        "gaussian": {
            "amplitude": {"max": 5, "min": 0.0, "vary": True, "value": 1},
            "center": {"max": 7113, "min": 7110, "vary": True, "value": 7112},
            "fwhmg": {"max": 5.5, "min": 0.1, "vary": True, "value": 1.5},
        }
    },
    {
        "gaussian": {
            "amplitude": {"max": 5, "min": 0.0, "vary": True, "value": 1},
            "center": {"max": 7114, "min": 7110, "vary": True, "value": 7113.6},
            "fwhmg": {"max": 5.5, "min": 0.1, "vary": True, "value": 1.5},
        }
    },
    {
        "gaussian": {
            "amplitude": {"max": 5, "min": 0.0, "vary": True, "value": 1},
            "center": {"max": 7117, "min": 7113, "vary": True, "value": 7115.6},
            "fwhmg": {"max": 5.5, "min": 0.1, "vary": True, "value": 1.5},
        }
    },
    {
        "gaussian": {
            "amplitude": {"max": 5, "min": 0.0, "vary": True, "value": 1},
            "center": {"max": 7119, "min": 7115, "vary": False, "value": 7117.6},
            "fwhmg": {"max": 5.5, "min": 0.1, "vary": True, "value": 1.5},
        }
    },
    {
        "gaussian": {
            "amplitude": {"max": 5, "min": 0.0, "vary": True, "value": 1},
            "center": {"max": 7121, "min": 7117, "vary": True, "value": 7119.6},
            "fwhmg": {"max": 5.5, "min": 0.1, "vary": True, "value": 1.5},
        }
    },
    {
        "gaussian": {
            "amplitude": {"max": 5, "min": 0.0, "vary": True, "value": 1},
            "center": {"max": 7125, "min": 7120, "vary": True, "value": 7121.6},
            "fwhmg": {"max": 5.5, "min": 0.1, "vary": True, "value": 1.5},
        }
    },
    {
        "cgaussian": {
            "amplitude": {"max": 1.3, "min": 0.1, "vary": True, "value": 0.83},
            "center": {"max": 7135, "min": 7123, "vary": True, "value": 7125},
            "fwhmg": {"max": 5, "min": 0.1, "vary": True, "value": 0.8},
        }
    },
]
spn.solver_model(initial_model=initial_model)
```

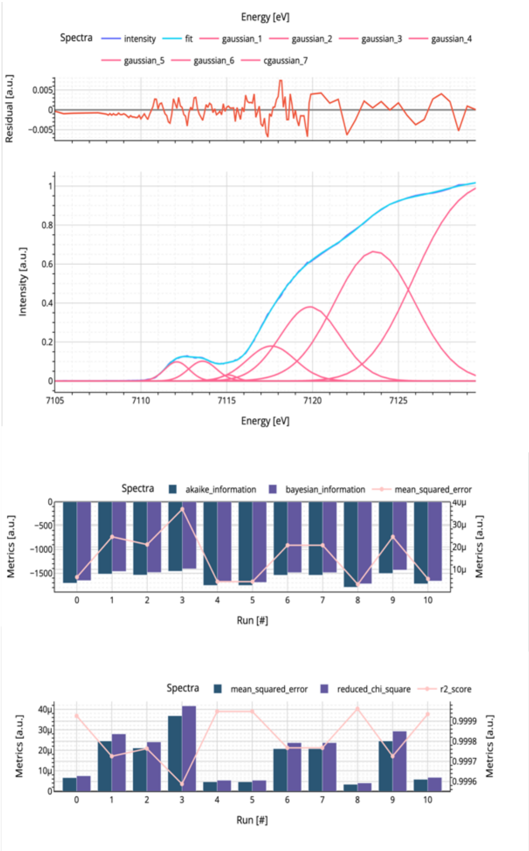

Figure S20. Run 10. Adapted with permission from Ref. 1. Copyright 2023 American Chemical Society.

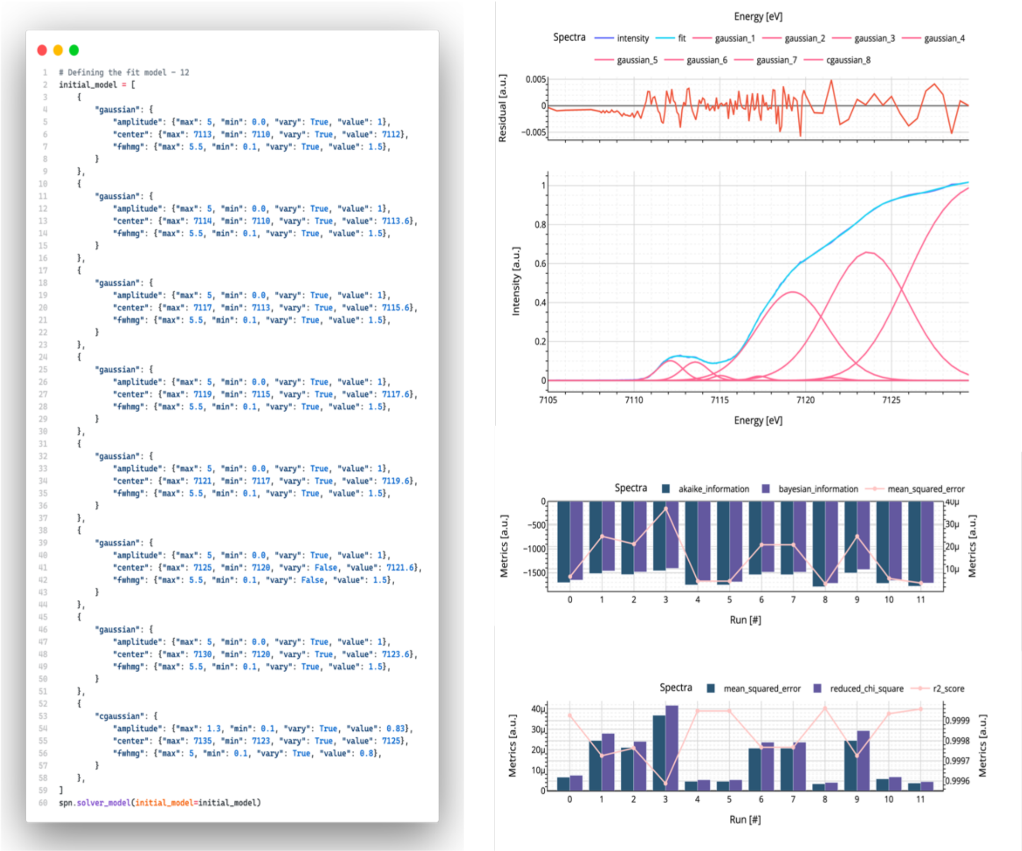

Figure S21. Run 11. Adapted with permission from Ref. 1. Copyright 2023 American Chemical Society.

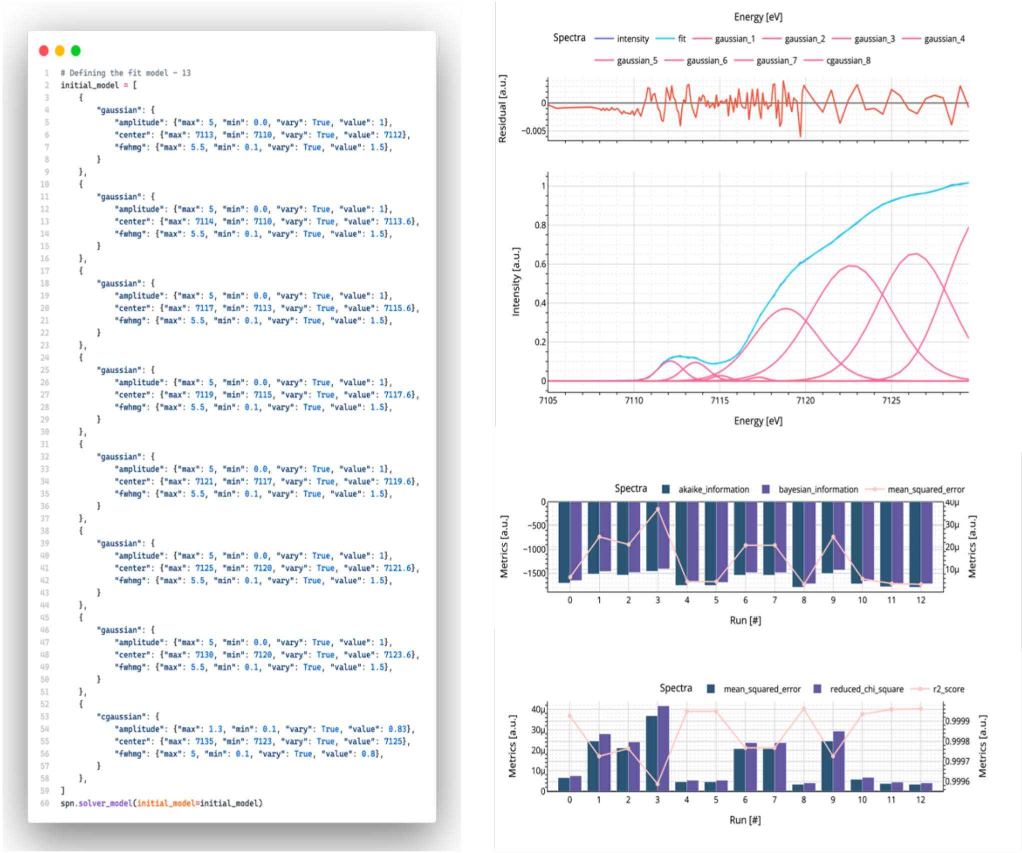

Figure S22. Run 12. Adapted with permission from Ref. 1. Copyright 2023 American Chemical Society.

References

(1) Yogendra, S.; Wilson, D. W. N.; Hahn, A. W.; Weyhermuller, T.; Van Stappen, C.; Holland, P.; DeBeer, S. Sulfur-Ligated [2Fe-2C] Clusters as Synthetic Model Systems for Nitrogenase. *Inorg Chem* **2023**, *62* (6), 2663-2671. DOI: 10.1021/acs.inorgchem.2c03693.
(2) *pandas-dev/pandas: Pandas*; Zenodo: 2023. https://doi.org/10.5281/zenodo.7979740.
(3) Newville, M.; Otten, R.; Nelson, A.; Ingargiola, A.; Stensitzki, T.; Allan, D.; Fox, A.; Carter, F.; Osborn, R.; Pustakhod, D. lmfit/lmfit-py: 1.0. 3. *Zenodo* **2021**. DOI: 10.5281/zenodo.598352. Newville, M.; Stensitzki, T.; Allen, D. B.; Rawlik, M.; Ingargiola, A.; Nelson, A. LMFIT: Non-linear least-square minimization and curve-fitting for Python. *Astrophysics Source Code Library* **2016**, ascl: 1606.1014. DOI: 10.5281/zenodo.598352.
(4) Hughes, I.; Hase, T. *Measurements and their Uncertainties: A practical guide to modern error analysis*; Oxford University Press, 2010.
(5) Field, A.; Miles, J.; Field, Z. *Discovering statistics using R*; SAGE Publications, 2012.
(6) Newville, M.; Stensitzki, T.; Otten, R.; et al. *Calculation of confidence intervals — Non-Linear Least-Squares Minimization and Curve-Fitting for Python*. 2023. https://lmfit.github.io/lmfit-py/confidence.html.
(7) Forschungsgemeinschaft, D. Guidelines for Safeguarding Good Research Practice. Code of Conduct. **2022**. DOI: 10.5281/zenodo.6472827.
(8) *Pydantic*. 2023. https://docs.pydantic.dev/latest/.
(9) Pedregosa, F.; Varoquaux, G.; Gramfort, A.; Michel, V.; Thirion, B.; Grisel, O.; Blondel, M.; Prettenhofer, P.; Weiss, R.; Dubourg, V.; et al. Scikit-learn: Machine Learning in Python. *Journal of Machine Learning Research* **2011**, *12* (85), 2825-2830.
(10) Dabbas, E. *Interactive Dashboards and Data Apps with Plotly and Dash: Harness the power of a fully fledged frontend web framework in Python - no JavaScript required*; Packt Publishing, 2021.
